# Supplementary material for: Geminin inhibits DNA replication licensing by sterically blocking CDT1-MCM2 interactions
Source: Nat Commun. 2025 Dec 9;16:11040. doi: 10.1038/s41467-025-67073-0 (PMC12695944; doi:10.1038/s41467-025-67073-0)
Supplement: Supplementary file 1 — Supplementary Information [file 41467_2025_67073_MOESM1_ESM.pdf]

SUPPLEMENTARY INFORMATION

Geminin inhibits DNA replication licensing by sterically blocking CDT1-MCM2 interactions

Joshua Tomkins[1,2,3*], Lucy V Edwardes[1,2*], Sarah V Faull[1,2], Matthew Peach[1,2], Peter J Gillespie[4,5], Vera Leber[1,2], Anna Schmidt[1,2], Halil Bounoua[1,2], Nicholas Sim[1,2], Rosa Camarillo1,[2], J Julian Blow[4,5], Alexis R Barr[1,2 #], Anna Barnard[3 #] and Christian Speck[1,2 #]

[1] Institute of Clinical Sciences, Faculty of Medicine, Imperial College London, London, W12 0HS, UK

[2] MRC Laboratory of Medical Sciences (LMS), London, W12 0HS, UK

[3] Department of Chemistry, Molecular Sciences Research Hub, Imperial College London, 82 Wood Lane, London, W12 0BZ, UK

[4] Division of Molecular, Cell and Developmental Biology, School of Life Sciences, University of Dundee, Dundee, DD1 5EH, UK.

[5] School of Biological Sciences, University of East Anglia, Norwich Research Park, Norwich NR4 7TJ, UK

* These authors contributed equally to the work.

#Correspondence:

a.barr@lms.mrc.ac.uk; a.barnard@imperial.ac.uk; chris.speck@imperial.ac.uk

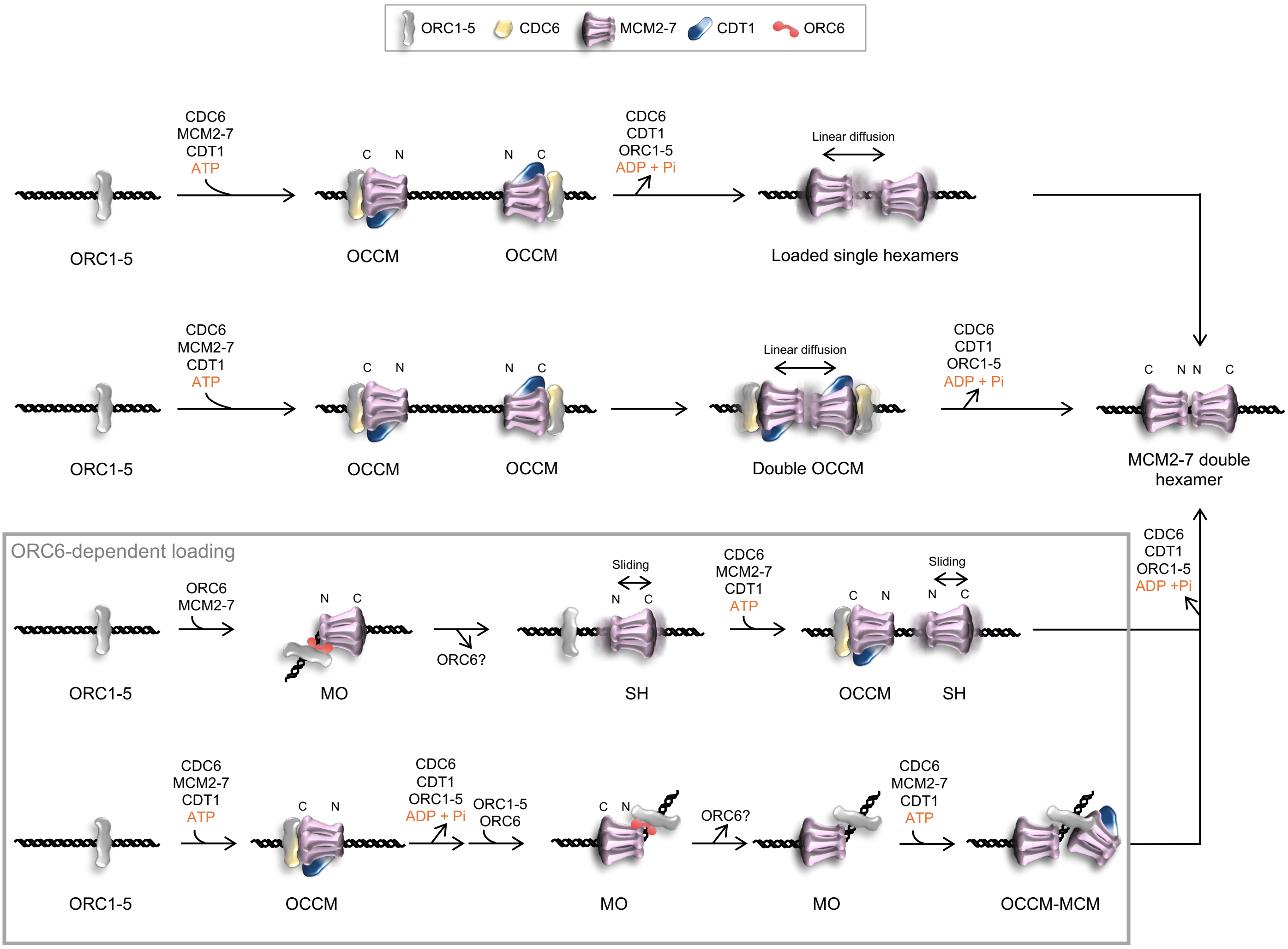

Supplementary Figure 1: Model of human MCM loading

Human MCM loading is proposed to occur through ORC6-independent and ORC6-dependent pathways. Helicase loading is more efficient with ORC6, therefore it is though to be the dominant pathway (shown in box). Figure based upon[1,2].

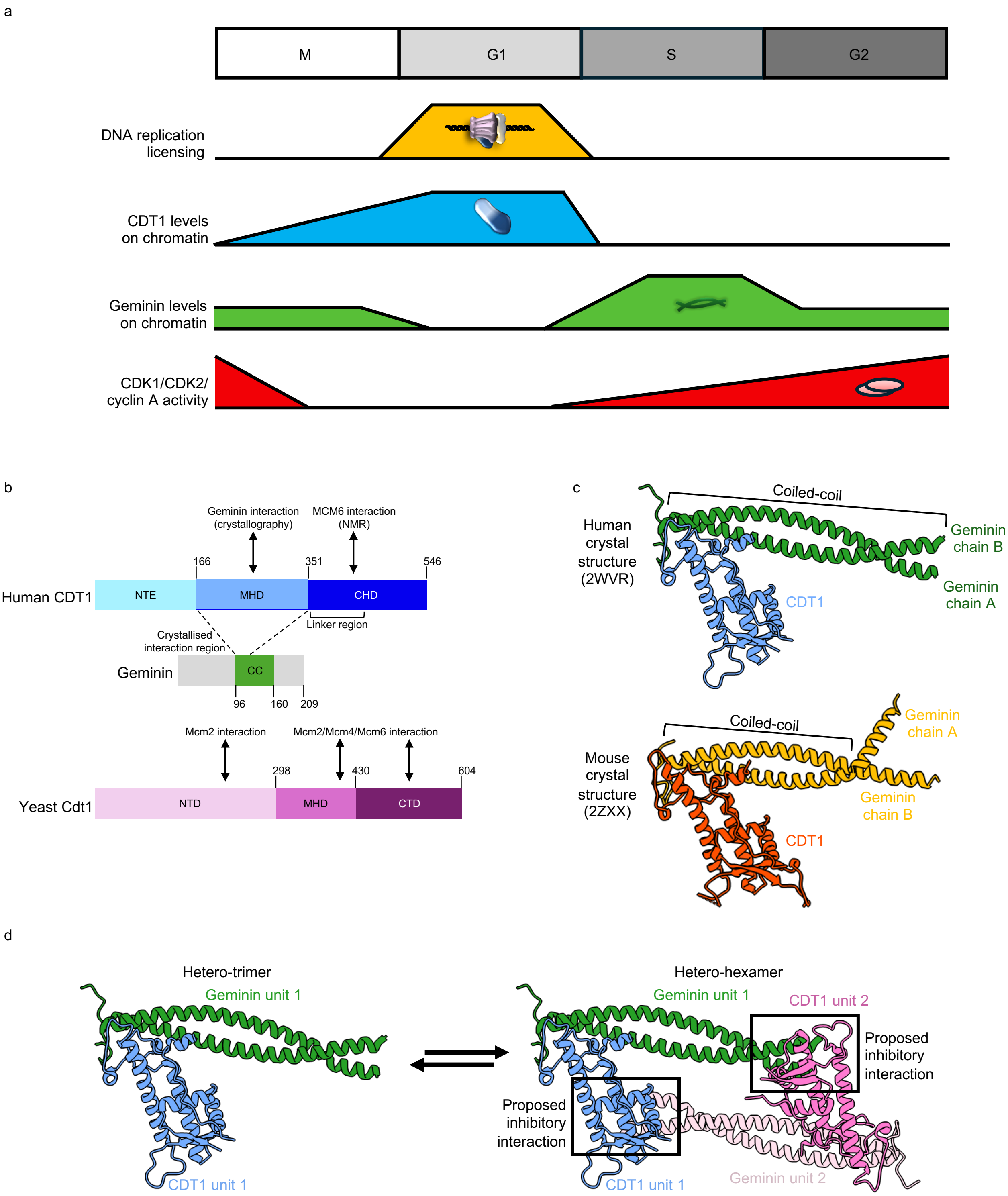
a
M
G1
S
G2
DNA replication licensing
CDT1 levels on chromatin
Geminin levels on chromatin
CDK1/CDK2/ cyclin A activity
b
Geminin interaction (crystallography)
MCM6 interaction (NMR)
166
351
546
Human CDT1
NTE
MHD
CHD
Linker region
Crystallised interaction region
Geminin
CC
96
160
209
Mcm2 interaction
Mcm2/Mcm4/Mcm6 interaction
298
430
604
Yeast Cdt1
NTD
MHD
CTD
c
Coiled-coil
Human crystal structure (2WVR)
Geminin chain B
Geminin chain A
CDT1
Coiled-coil
Geminin chain A
Mouse crystal structure (2ZXX)
Geminin chain B
CDT1
d
Hetero-trimer
Geminin unit 1
CDT1 unit 1
Hetero-hexamer
Geminin unit 1
CDT1 unit 2
Proposed inhibitory interaction
Proposed inhibitory interaction
CDT1 unit 1
Geminin unit 2

Supplementary Figure 2: CDT1 interacts with both geminin and MCM2-7

a Schematic of the timing DNA licensing in relation to CDT1 and geminin levels on chromatin and CDK1/CDK2/cyclin A activity during the cell cycle. Figure based on[3,4]. **b** Domain structure of human CDT1 (blue) showing that the middle helical domain (MHD) interacts with the geminin coiled-coil (PDB ID 2WVR), and MCM2-7 interacts with the C-terminal helical domain (CHD) (PDB ID 2LE8) but not the N-terminal extension (NTE). The crystalised interaction region from structure 2WVR is shown by the dashed lines. In yeast, Cdt1 (purple) interacts with Mcm subunits across all three of its domains. NTD = N-terminal domain, CTD = C-terminal domain. **c** Top: human crystal structure (PDB ID 2WVR), of the geminin coiled-coil (green, formed by dimerization of residues 96-160 from geminin chain A and geminin chain B) and NTE of human CDT1 (blue, residues 167-252, 268-353) showing the interaction highlighted by the dashed lines in part (**b**). Bottom: the mouse CDT1-geminin crystal structure (PDB ID 2ZXX) forms a similar interaction to the human structure. CDT1 is shown in red with geminin in orange. **d** The CDT1-geminin hetero-hexamer forms through the dimerisation of the CDT1-Geminin hetero-trimer via a binding interaction between CDT1 and the C-terminus of the geminin coiled-coil (highlighted by boxes). It is proposed that the structures exist in equilibrium, where the hetero-trimer is a permissive (does not inhibit CDT1) and the hetero-hexamer is inhibitory towards CDT1 and DNA licensing (PDB ID 2WVR). Figures made using Chimera X.

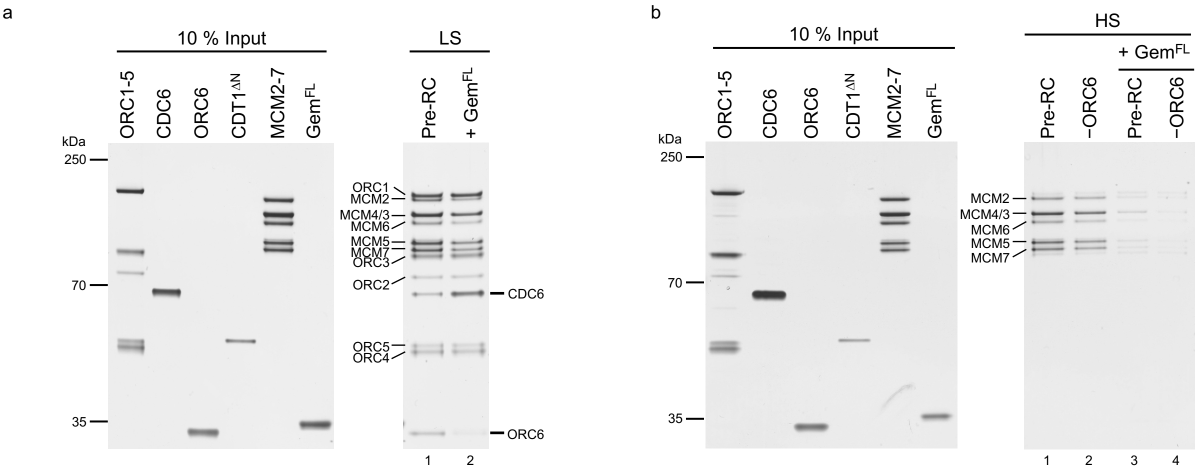

Supplementary Figure 3: The role of CDC6 and ORC6 in pre-RC formation

a CDC6 is stabilised even when used at 50% of the amount used in the standard pre-RC assay. **b** Geminin can inhibit MCM2-7 loading in both ORC6-dependent and ORC6-independent pre-RC pathways. Representative of three biological repeats. Source data are provided as a Source Data file.

a

Pre-RC control

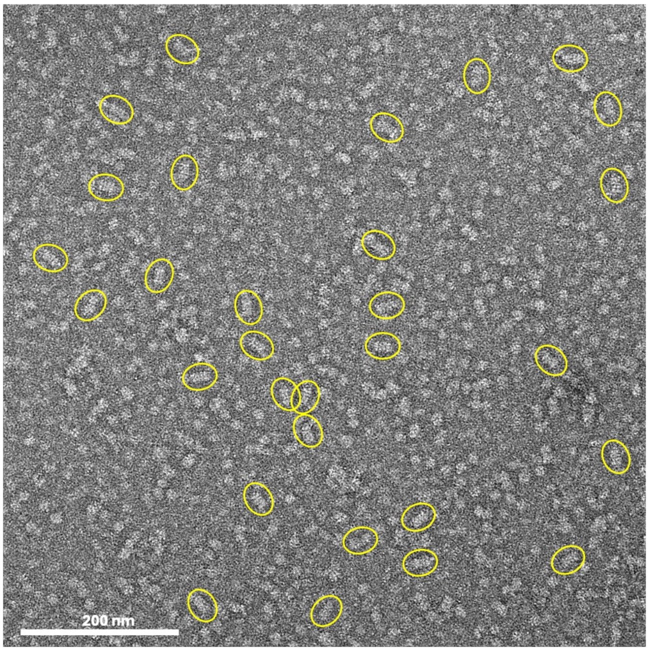

b

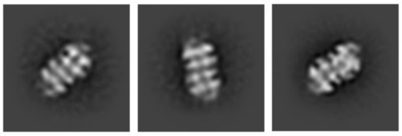

c

Number of micrographs	DH count	DH/micrograph	Mean
1038	33414	32.19	36.63
598	24555	41.06	

d

+Gem^{FL}

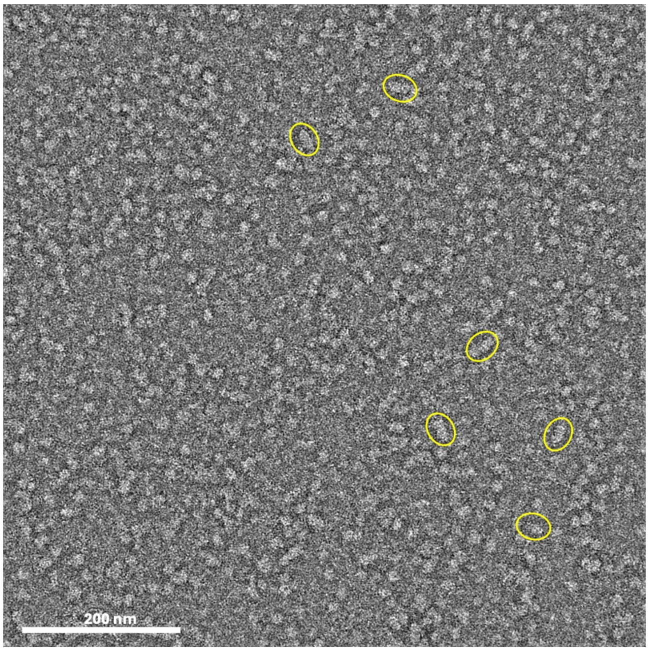

e

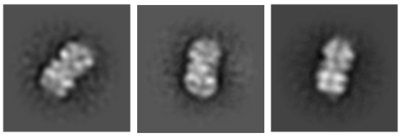

f

Number of micrographs	DH count	DH/micrograph	Mean
583	2620	4.49	6.34
647	4948	7.65	

g

+Gem^{FL}–CDT1$^{\Delta N}$

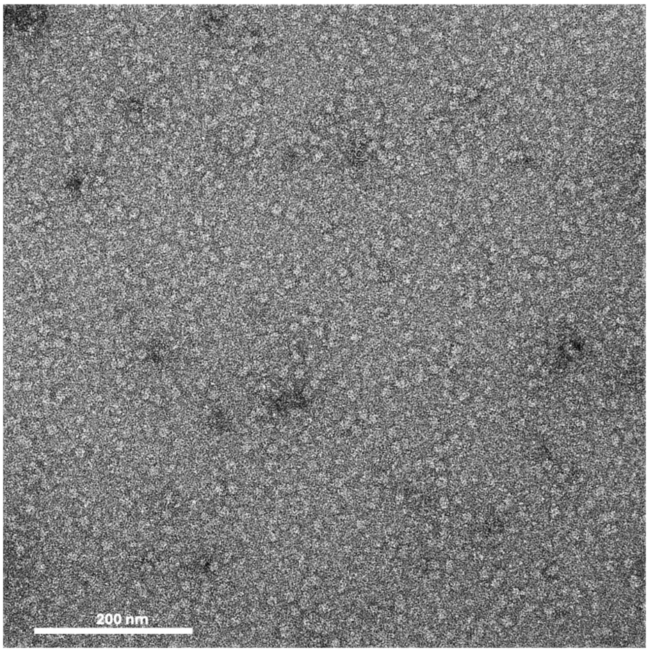

h

Number of micrographs	DH count	DH/micrograph	Mean
1044	0	0	0
547	0	0	

Supplementary Figure 4: In solution negative stain EM analysis of double hexamer (DH) formation in the presence of geminin

a Representative micrograph of the control pre-RC reaction. An average of ~37 DHs (circled in yellow) per micrograph were counted across two datasets, representing 1600 micrographs. **b** Representative class averages of 2D DH classes. **c** Table detailing the two datasets collected for the control pre-RC condition. **d** Representative micrograph of the pre-RC reaction in the presence of geminin. An average of ~6 DHs (circled in yellow) per micrograph were counted across two datasets, representing 1200 micrographs. **e** Representative 2D 2D class averages of DH classes from the plus geminin reaction. Due to the lower particle number the resolution of classes is lower than the control, but the distinctive DH shape is still visible. **f** Table detailing the two datasets collected for the pre-RC plus geminin condition. **g** Representative micrograph of the pre-RC reaction minus CDT1 in the presence of geminin. No double hexamers were observed across two datasets, representing 1600 micrographs. **h** Table detailing the two datasets collected for the minus CDT1 and geminin condition.

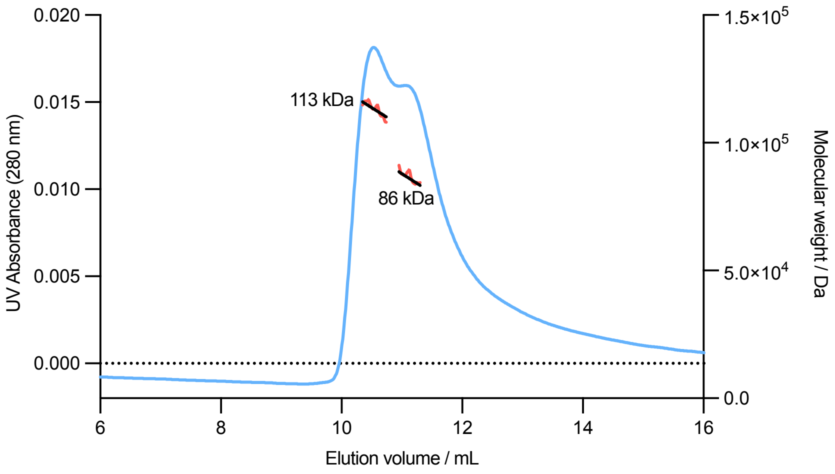

Supplementary Figure 5: Determining oligomerisation states of CDT1-geminin using SEC-MALS

SEC-MALS elution of co-purified $CDT1^{FL}$-geminin at 2.6 µM. Two peaks were resolved, one with a molecular weight (M_W) corresponding to 113 kDa, which is consistent with hetero-trimer (calculated M_W = 115 kDa) and one peak of unknown identity at 86 kDa. No peaks corresponding to a possible $CDT1^{FL}$-geminin hetero-hexamer (calculated M_W = 229 kDa) were visible. Data representative of two independent experiments. Source data are provided as a Source Data file.

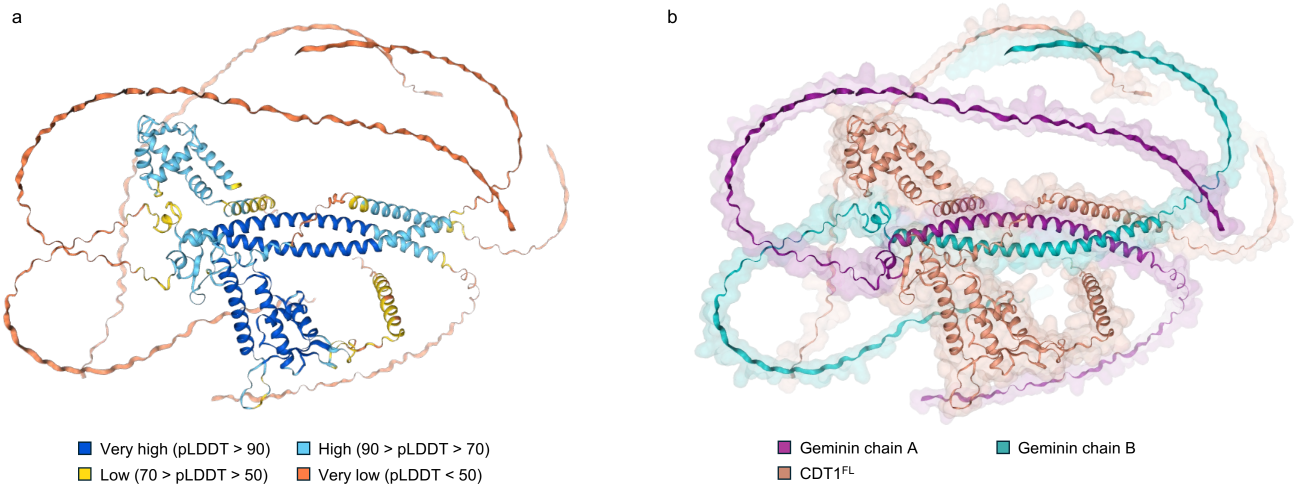

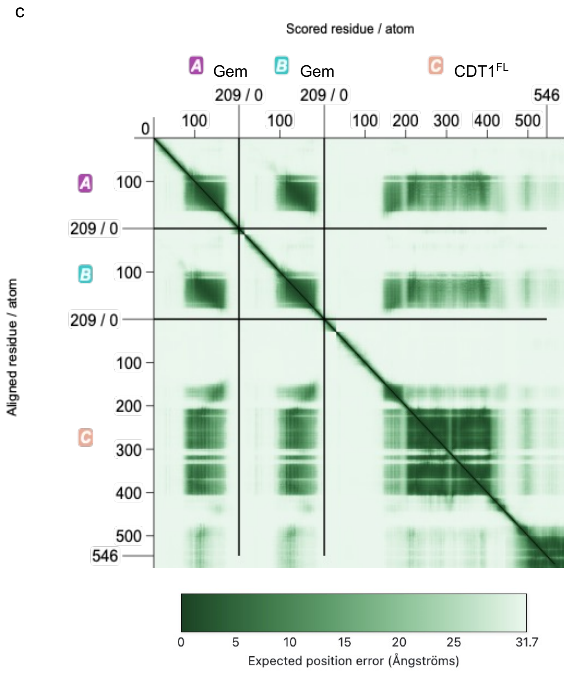

d

Region	CDT1 residues	Geminin A residues	Geminin B residues
Region 1	113-150	132-157	143-160
Region 2	434-446	98-114	98-114
Region 3.1	-	96-160	96-160
Region 3.2	318-333	76-95	-

e

Region	CDT1:geminin A contact probability	CDT1:geminin B contact probability	CDT1:geminin A PAE	CDT1:geminin B PAE
Region 1	0.07	0.06	14.11	13.56
Region 2	0.07	0.06	6.71	7.39
Region 3.1	0.13	-	9.43	-
	Geminin A:geminin B contact probability		Geminin A:geminin B PAE	
Region 3.2	0.28		6.81	

Supplementary Figure 6: AlphaFold reveals new interactions between CDT1 and geminin

a AlphaFold3 model of the CDT1^{FL}-geminin hetero-trimer coloured by pLDDT score. Ranking score = 0.65, predicted template modelling (pTM) = 0.39, interface PTM (iPTM) = 0.35. **b** CDT1^{FL}-geminin complex from part (**a**) coloured by chain. **c** Predicted alignment error (PAE) plot of the AlphaFold model, produced using PAE Viewer. **d** Residue numbers of interaction regions observed between CDT1^{FL} and geminin in the AlphaFold prediction. **e** Contact probabilities of the interacting residues defined in part (**d**). Values were extracted from the .JSON output files using a Python script. Values for all chain C (CDT1^{FL}) residues in the interaction were extracted for the defined regions of geminin chains A and B. The average of non-zero values were calculated for the contact probabilities to remove non-interacting residues, such as those on the opposite side of the helix to the interaction. Source data are provided as a Source Data file.

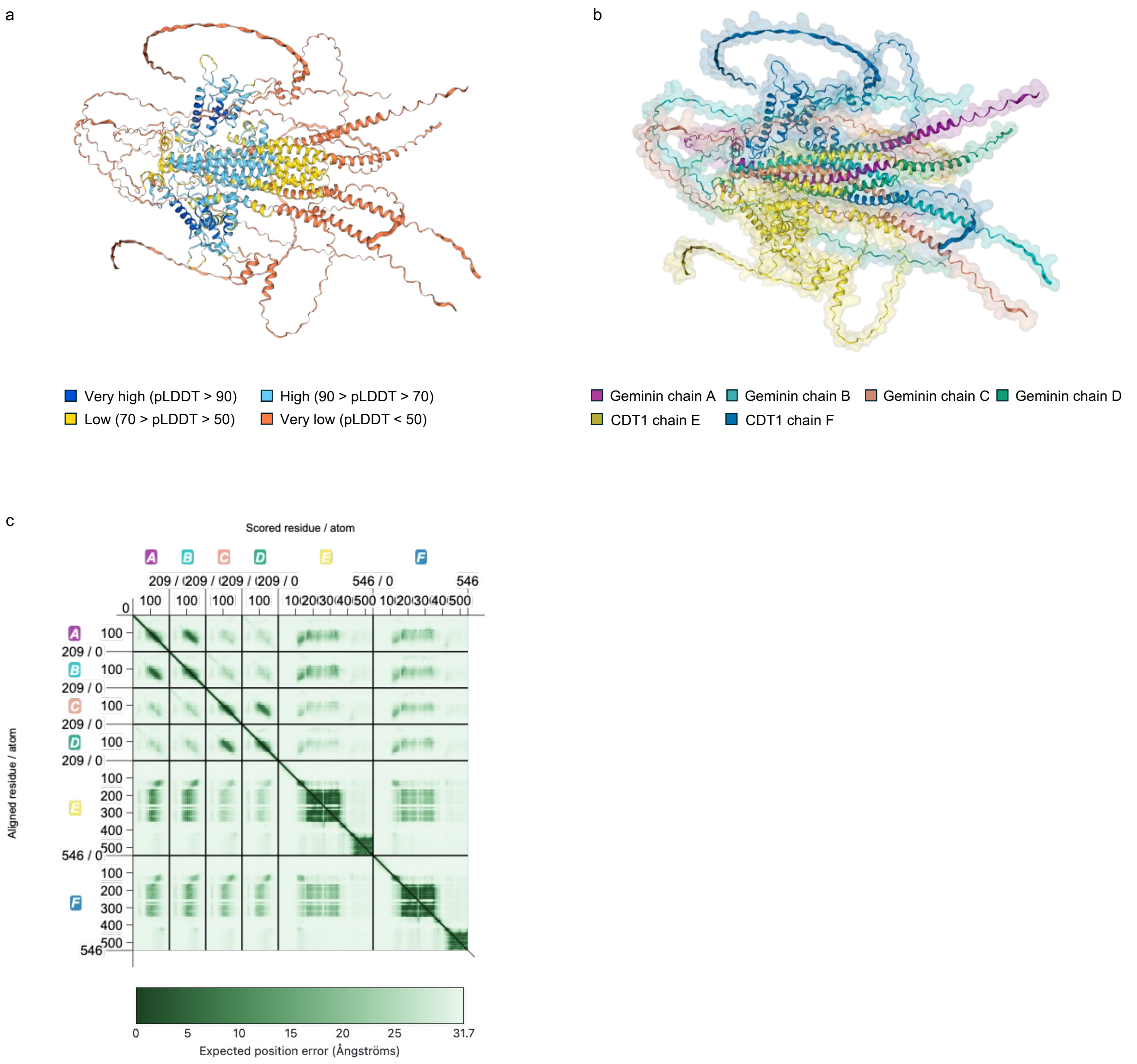

Supplementary Figure 7: AlphaFold3 prediction of the CDT1^{FL}-geminin hetero-hexamer

a Highest-ranking AlphaFold3 model of the CDT1^{FL}-geminin hetero-hexamer coloured by pLDDT score. Ranking score = 0.52, predicted template modelling (pTM) = 0.32, interface PTM (iPTM) = 0.27. **b** CDT1^{FL}-geminin complex from (**a**) coloured by chain. **c** Predicted alignment error (PAE) plot of the AlphaFold model**.** Chains A-D = geminin, chains E and F = CDT1^{FL}. Figure produced using PAE Viewer.

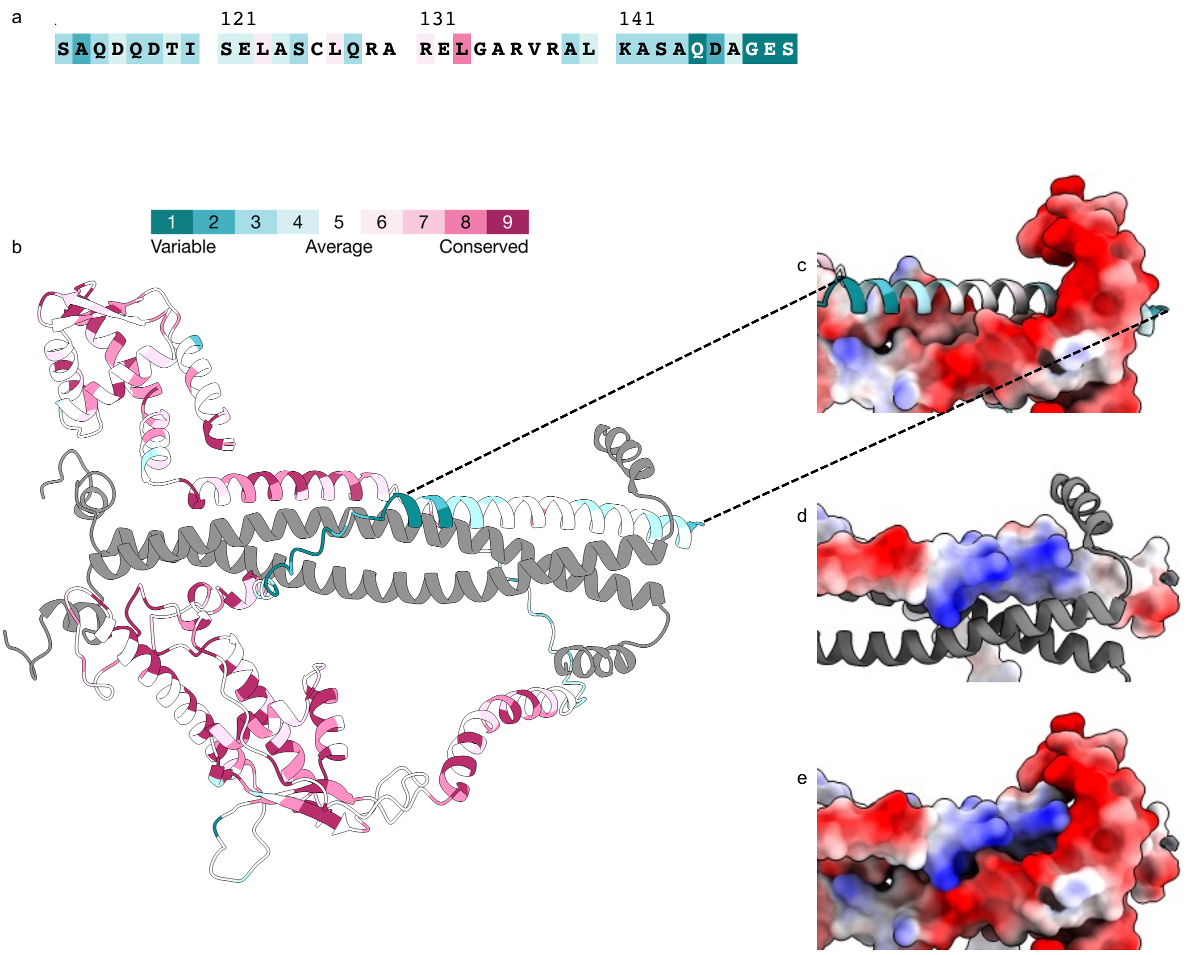

Supplementary Figure 8: ConSurf analysis of CDT1^{FL}

a Sequence of CDT1 alpha-helix (residues 113-150) predicted to interact with geminin in region 1 of our AlphaFold3 model. Residues are coloured using the ConSurf colour key in part (**b**). **b** AlphaFold3 model of CDT1^{FL} and geminin (grey). CDT1 is coloured by residue conservation according to results generated by the ConSurf webserver[5]. The unstructured regions of CDT1 and geminin are not shown. **c** Zoomed-in view of helix 113-150 with geminin coloured by electrostatic potential to highlight the negatively-charged region (red) interacting with the CDT1 helix. **d** view (**c**) with geminin (grey) shown in cartoon form and CDT1 coloured by electrostatic potential. **e** Both geminin and CDT1 shown by electrostatic potential to highlight the suspected charge-driven interaction between helix 113-150 and C-terminus of the geminin coiled-coil. Figures made using Chimera.

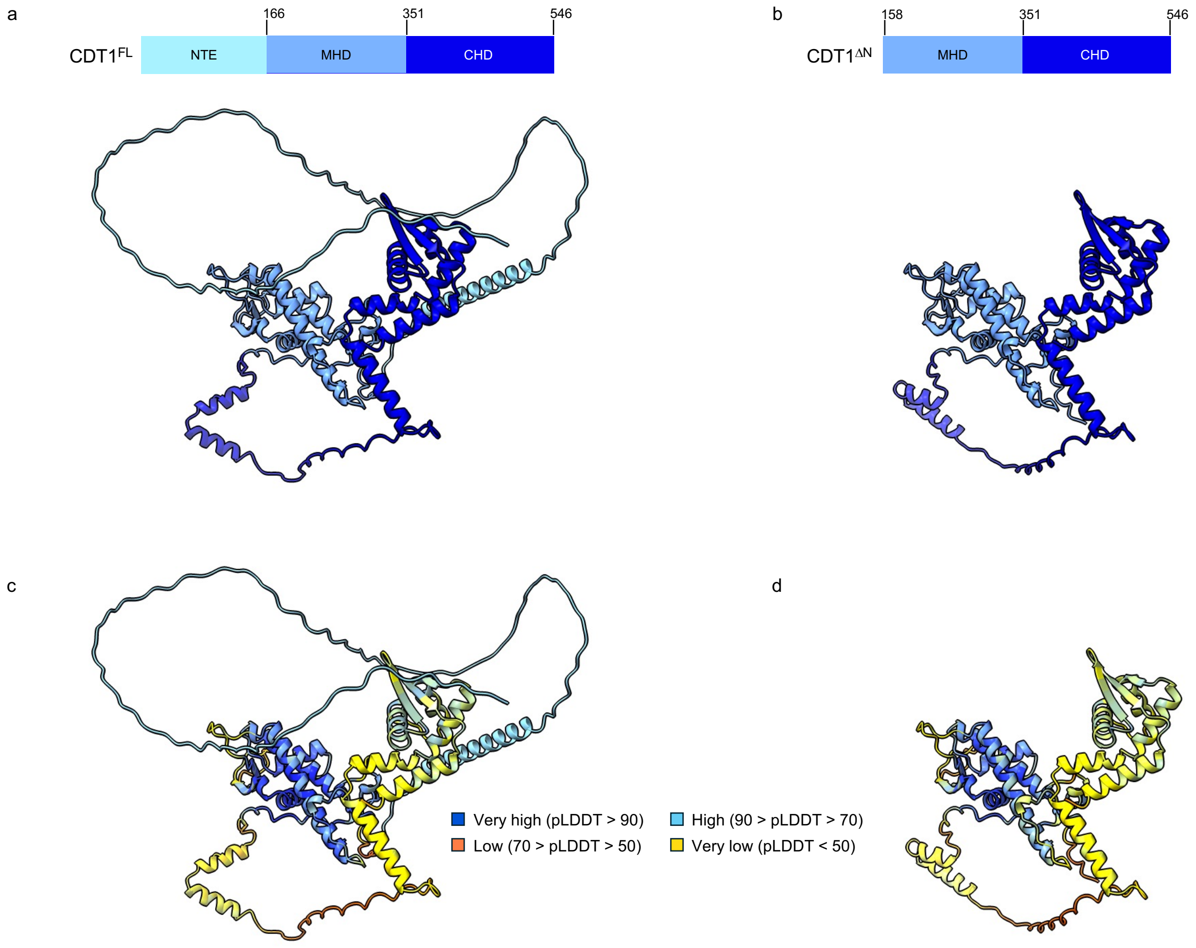

Supplementary Figure 9: The N-terminus of human CDT1 is unstructured

AlphaFold3 models of **a** full-length CDT1 (CDT1^{FL}) and **b** variant (CDT$^{\Delta N}$) with truncation of the unstructured N-terminal extension (NTE). MHD = middle helical domain, CHD = C-terminal helical domain. **c** CDT1^{FL} AlphaFold3 model coloured by pLDDT score. **d** CDT1$^{\Delta N}$ AlphaFold3 model coloured by pLDDT score. Figures made using Chimera X.

a

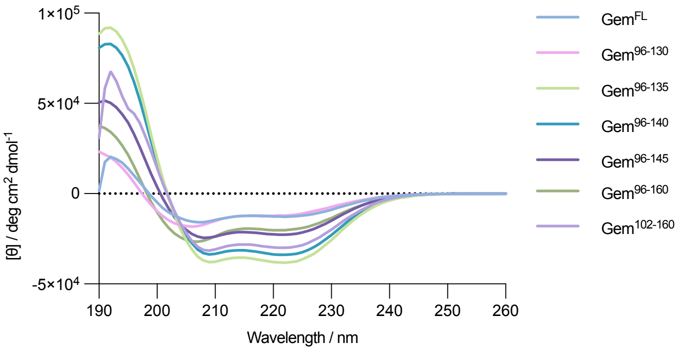

b

Peptide	Helicity / %
Gem^{FL}	31
Gem^{96-130}	38
Gem^{96-135}	95
Gem^{96-140}	90
Gem^{96-145}	68
Gem^{96-160}	60
$Gem^{102-160}$	75

Supplementary Figure 10: CD spectra and helicity of geminin coiled-coil mimetic peptides

a Coiled-coil mimetic peptides CD spectra of ~ 50 μM of peptide in 10 mM phosphate buffer at pH 7.4. **b** Helicities of peptides, calculated using the K2D3 webserver[6]. As the geminin coiled-coil is helical, perfect mimicry would result mimetic peptides displaying 100 % helicity. Source data are provided as a Source Data file.

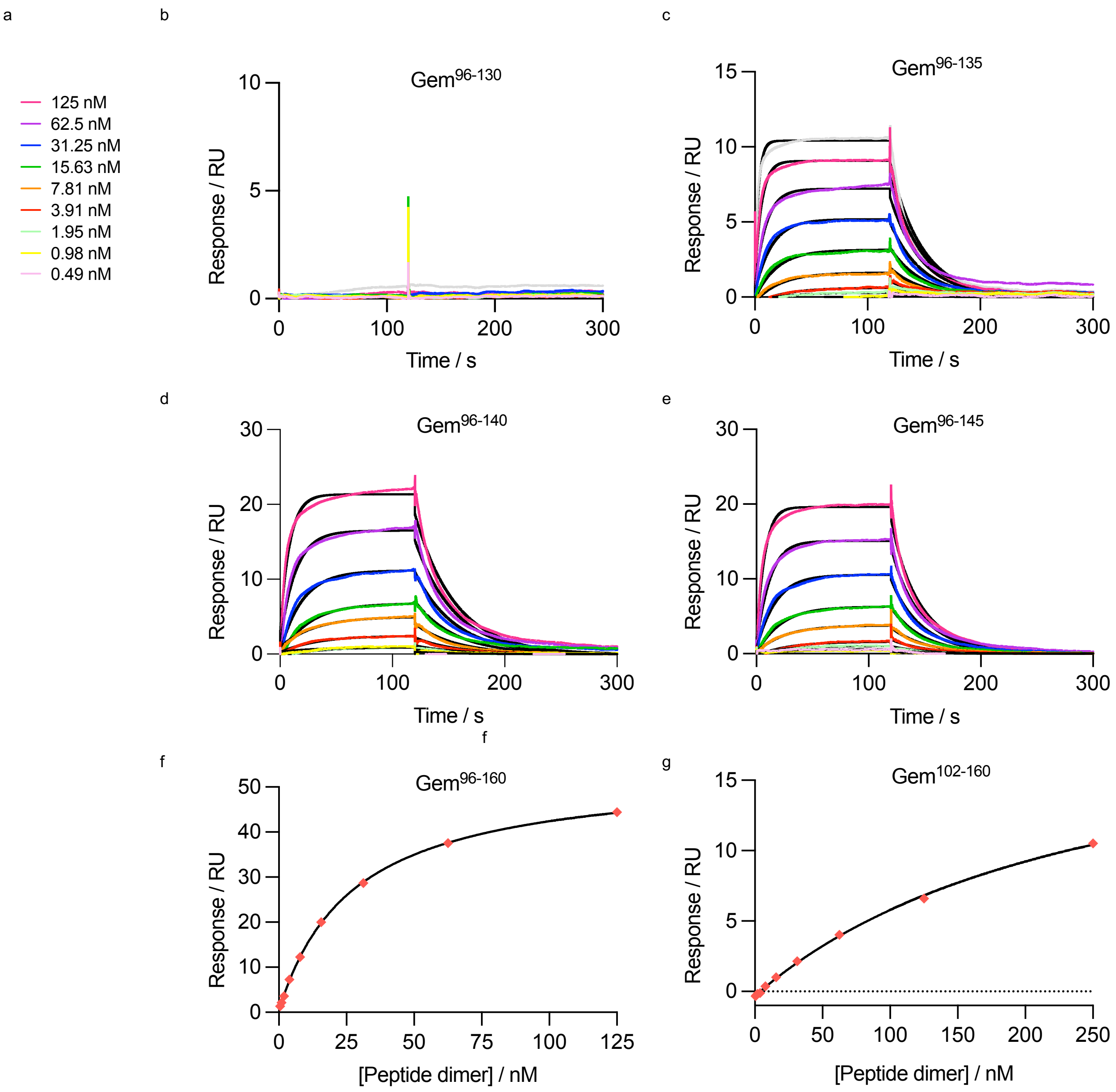

Supplementary Figure 11: SPR experiments for CDT1$^{\Delta N}$ and coiled-coil mimetic peptides

a Key showing concentrations of coiled-coil mimetic peptide dimers. **b-e** Representative multi-cycle kinetics sensorgrams of coiled-coil peptides binding to CDT1$^{\Delta N}$. Raw data is shown in colour, with kinetic fits in black. Not all concentrations were used for all kinetic models. **f-g** Suitable kinetic fits could not be modelled for Gem$^{96-160}$ and Gem$^{102-160}$, therefore affinity modelling was used. Source data are provided as a Source Data file.

a

Peptide	Mw (calc) / Da	Mw (SEC-MALS) / Da	Oligomerisation state
Gem^{FL}	23433.95	65000 ± 3000	2.77 ± 0.11
$Gem^{96\text{-}130}$	4402.95	5210 ± 80	1.18 ± 0.02
$Gem^{96\text{-}135}$	4984.71	11160 ± 150	2.24 ± 0.03
$Gem^{96\text{-}140}$	5613.39	14000 ± 600	2.49 ± 0.11
$Gem^{96\text{-}145}$	6096.96	14500 ± 500	2.38 ± 0.08
$Gem^{102\text{-}160}$	7059.09	15800 ± 400	2.23 ± 0.06

b

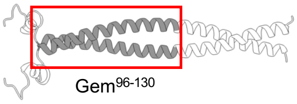

c

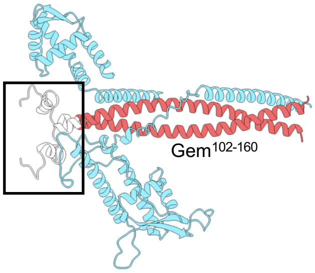

Supplementary Figure 12: Molecular weights and oligomerisation states of geminin coiled-coil mimetic peptides

a Molecular weights (M_W) were determined using SEC-MALS and are displayed as the the mean of 3 independent experiments ±SE. Oligomerisation states were determined by dividing the experimentally determined M_W with the calculated M_W. **b** Cartoon of geminin residues 76-160, highlighting the region covered by peptide $Gem^{96\text{-}130}$, which is unable to dimerise due to truncation of the coiled-coil resulting in an oligomerisation state of 1.18. **c** Cartoon of geminin residues 76-160 and $CDT1^{FL}$ (light blue), highlighting the region covered by peptide $Gem^{102\text{-}160}$ (red), which is unable to bind CDT1 due to truncation of the N-terminal region (residues 76-101, shown in the box). Structural figures made using Chimera X. Source data are provided as a Source Data file.

a

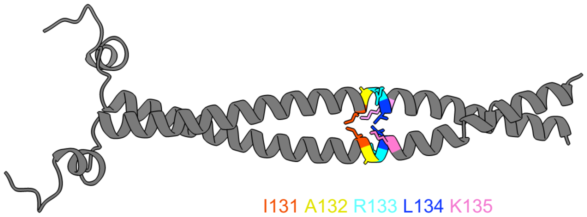

b

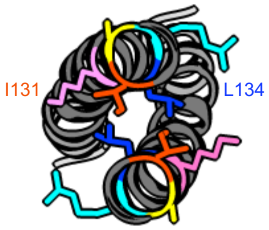

c

Peptide	Mw (calc) / Da	Mw (SEC-MALS) / Da	Oligomerisation state
Gem$^{76\text{-}145}$	8286.43	17400 ± 300	2.10 ± 0.04
Gem^{I131A}	8244.34	15130 ± 60	1.84 ± 0.01
Gem^{L134A}	8244.34	16250 ± 150	1.97 ± 0.02
Gem$^{I131A, L134A}$	8202.27	12000 ± 400	1.46 ± 0.05

d

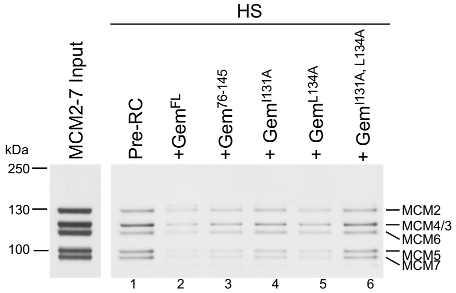

e

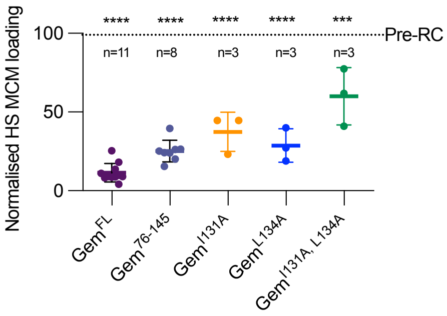

Supplementary Figure 13: Disrupting dimerisation of the geminin coiled-coil using point mutations

a Side and **b** cross-section view of an AlphaFold3 model of geminin residues 76-160. The side chains of residues 131-135 are shown to illustrate that I131 (orange) and L134 (royal blue) are between the coiled-coil helices in a hydrophobic space. The interaction between the residues may help to stabilise the coiled-coil dimer. Figure made using Chimera X. **c** Peptide molecular weights (M_W, shown as the mean ±SE of three experiments) were determined using SEC-MALS. Oligomerisation states were determined by dividing the experimentally determined M_W by the calculated M_W. **d** High-salt (HS) washed elutions from a pre-RC assay after addition of Gem^{FL}, Gem$^{76\text{-}145}$ or Gem$^{76\text{-}145}$ with mutations designed to disrupt dimerisation. **e** Quantification of the mean ±SD of MCM2-7 band intensity in part (**d**) relative to the control pre-RC reaction. Data was compared to the control pre-RC reaction using ordinary one-way ANOVA. **** indicates P<0.0001, ***P=0.0003. Source data are provided as a Source Data file.

a

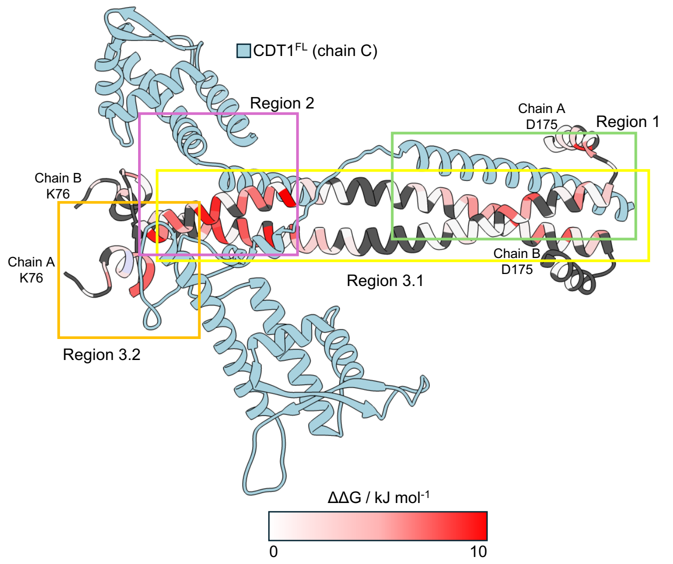

b

Geminin interactions with CDT1FL			
Geminin residue	Geminin chain A ΔΔG / kJ mol -1	Geminin chain B ΔΔG / kJ mol -1	Region
V81	3.3	0.8	Region 3.2
L88	2.9	0.0	Region 3.2
M89	5.5	0.1	Region 3.2
I90	5.6	3.2	Region 3.2
E92	3.6	0.9	Region 3.2
Y98	8.1	1.1	Region 2; Region 3.1
W99	4.3	0.6	Region 2; Region 3.1
E101	5.3	0.0	Region 2; Region 3.1
V102	2.2	0.4	Region 2; Region 3.1
K105	8.3	3.2	Region 2; Region 3.1
R106	6.3	6.0	Region 2; Region 3.1
R107	7.8	7.1	Region 2; Region 3.1
L110	5.0	5.9	Region 2; Region 3.1
Y111	2.3	4.5	Region 2; Region 3.1
E112	4.8	0.4	Region 2; Region 3.1
L114	4.5	8.1	Region 2; Region 3.1
E116	9.3	0.2	Region 3.1
N117	0.1	5.5	Region 3.1
L141	3.6	0.0	Region 1; Region 3.1
E143	2.5	0.0	Region 1; Region 3.1
V144	3.8	0.0	Region 1; Region 3.1
E146	0.0	5.1	Region 1; Region 3.1
H147	6.5	0.0	Region 1; Region 3.1
V148	0.2	2.7	Region 1; Region 3.1
Q149	0.0	6.0	Region 1; Region 3.1
Y150	4.4	0.0	Region 1; Region 3.1
L154	5.8	0.0	Region 1; Region 3.1
I155	0.4	4.2	Region 1; Region 3.1
E156	0.0	2.7	Region 1; Region 3.1
L158	3.3	0.2	Region 3.1
L163	0.0	3.3	
F166	0.0	7.3	

c

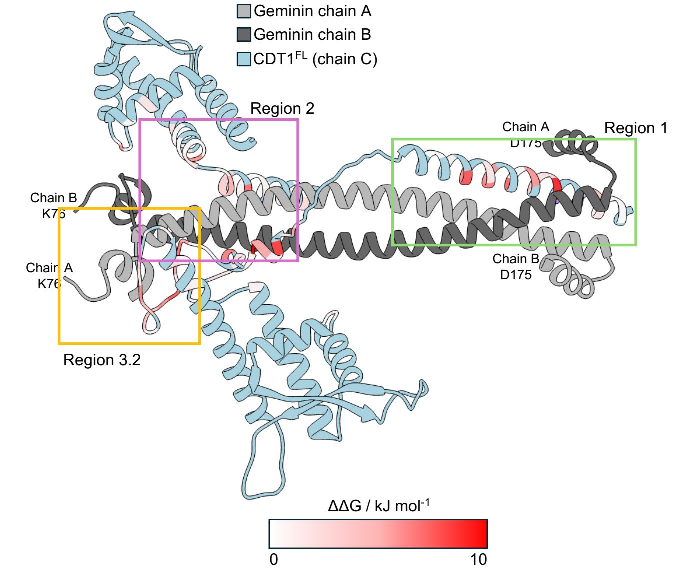

d

CDT1FL interactions with geminin		
CDT1FL residue	ΔΔG / kJ mol -1	Region
I120	2.2	Region 1
E122	3.6	Region 1
L123	8.4	Region 1
L127	8.7	Region 1
R129	2.1	Region 1
R131	4.1	Region 1
L133	6.0	Region 1
R136	4.1	Region 1
V137	5.1	Region 1
L140	4.5	Region 1
K141	6.8	Region 1
K166	3.7	
Y170	7.8	
R172	3.5	
F173	10.9	
I176	5.0	
P182	3.6	
V323	4.3	Region 3.2
P324	2.3	Region 3.2
Q327	2.2	Region 3.2
L328	2.3	Region 3.2
T329	4.3	Region 3.2
R330	6.6	Region 3.2
H332	3.3	Region 3.2
P333	5.6	Region 3.2
R334	3.4	
L435	5.8	Region 2
T439	2.9	Region 2
E445	4.2	Region 2
I489	4.9	

Supplementary Figure 14: Computational alanine scan indicating hotspot residues that contribute towards binding in the CDT1^{FL}:geminin hetero-trimer

a AlphaFold structure of CDT1^{FL}-geminin with geminin residues coloured according to BAlaS-predicted ΔΔG values (white to red). The boxes indicate the regions of interest identified in both the AlphaFold model and by greatest change ΔΔG (deepest red residues). CDT1^{FL} was designated as the ligand chain with geminin chains A and B as the receptors. **b** ΔΔG values for residues within at least one geminin chain that were predicted to have a ΔΔG of > 2.0 kJ mol^{-1} upon mutation to alanine. The regions of interest corresponding to the residues are shown in the last column, with colours matched to the boxes in part (**a**). **c** AlphaFold structure with CDT1^{FL} residues coloured according to BAlaS-predicted ΔΔG values. Geminin chains A and B were designated as ligands and CDT1^{FL} as the receptor. **d** ΔΔG values for CDT1^{FL} residues that were predicted to have a ΔΔG of > 2.0 kJ mol^{-1} upon mutation to alanine. Figures made using Chimera with scripts produced by the BUDE Alanine Scan webserver[7].

a

Peptide	Mw (calc)/Da	Major peak Mw/Da	Oligo. state	Minor peak Mw/Da	Oligo. state
Gem^{FL}	23433.95	65000 ± 3000	2.77 ± 0.11	n/a	n/a
$Gem^{96\text{-}145}$	6096.96	14500 ± 500	2.38 ± 0.08	n/a	n/a
$Gem^{90\text{-}145}$	6765.73	12200 ± 3000	1.80 ± 0.04	7600 ± 700	1.12 ± 0.11
$Gem^{82\text{-}145}$	7717.75	16500 ± 300	2.13 ± 0.04	10800 ± 600	1.40 ± 0.08
$Gem^{76\text{-}145}$	8286.43	17400 ± 300	2.10 ± 0.04	10600 ± 600	1.28 ± 0.07
$recGem^{76\text{-}145}$	8399.53	19300 ± 100	2.30 ± 0.02	n/a	n/a

b

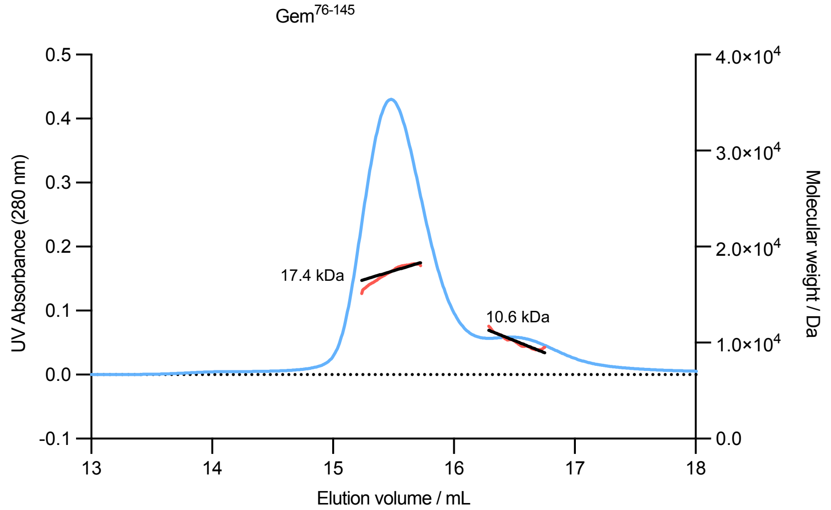

c

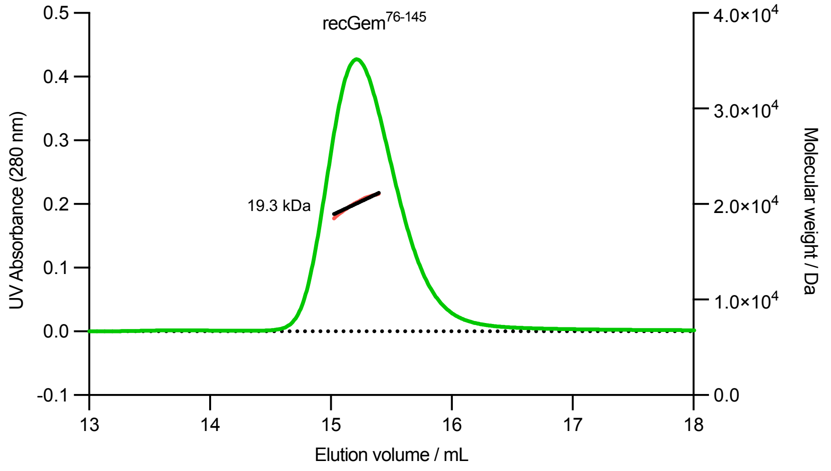

Supplementary Figure 15: Determining oligomerisation states of N-terminally extended peptides using SEC-MALS

a Table containing SEC-MALS determined molecular weights (M_W) and oligomerisation states (oligo. state). Data shown as the mean and SE of three measurements. **b** Representative SEC-MALS trace of peptide $Gem^{76\text{-}145}$. A major, predominantly dimer peak and a minor peak of predominantly monomeric material were present. **c** SEC trace of $recGem^{76\text{-}145}$ containing a predominantly dimer only peak. The measured weight-averaged MW (red) and the Zimm model fit (black) is shown for all peaks. Source data are provided as a Source Data file.

a

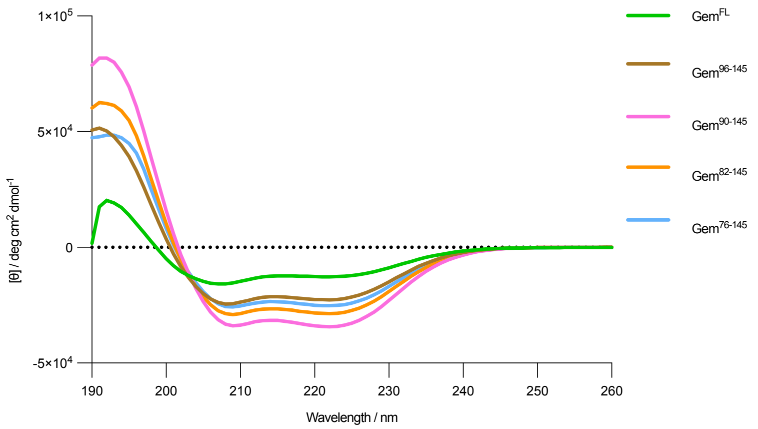

b

Peptide	Helicity / %
Gem^{FL}	31
$Gem^{96\text{-}145}$	68
$Gem^{90\text{-}145}$	90
$Gem^{82\text{-}145}$	78
$Gem^{76\text{-}145}$	70

Supplementary Figure 16: CD spectra and calculated helicities of N-terminally truncated peptides

a CD spectra of ~50 μM of peptide dissolved in 10 mM phosphate buffer at pH 7.4. **b** Helicities of peptides, as determined using the K2D3 webserver[6]. Extension of $Gem^{96\text{-}145}$ to $Gem^{90\text{-}145}$ increased helicity, but further extensions caused a decrease helicity suggesting that residues 90-95 may stabilise the coiled-coil helix. Further extension to $Gem^{82\text{-}145}$ then $Gem^{76\text{-}145}$ decreased the calculated helicities to 78 % and 70 % respectively. Source data are provided as a Source Data file.

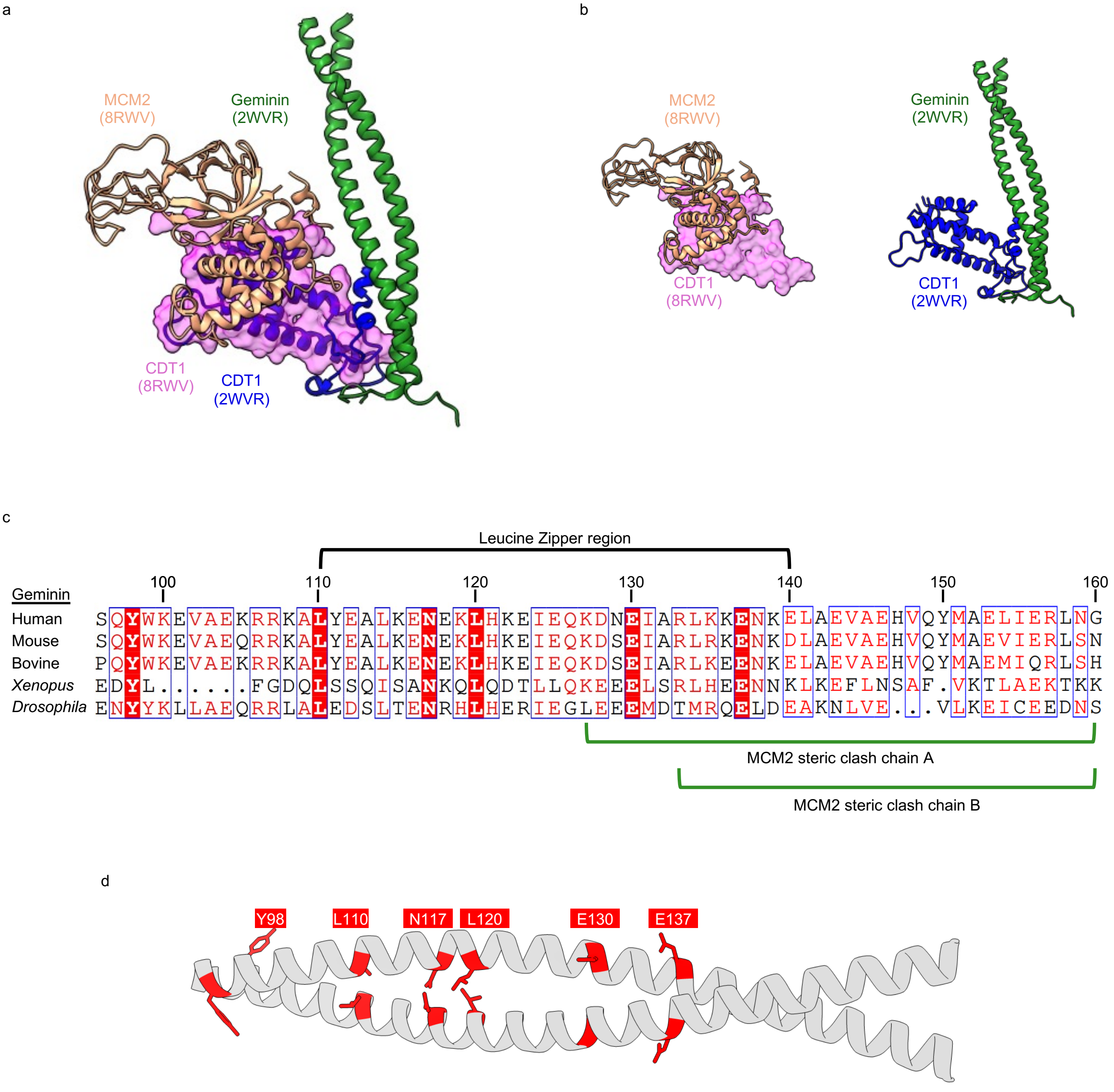

Supplementary Figure 17: How can the CDT1-geminin interaction block the CDT1-MCM2-7 interaction?

a N-terminal domain of MCM2 (peach cartoon) and middle-helical domain of CDT1 (pink surface view) from the human OCCM cryo-EM structure (PDB ID 8RWV) aligned by CDT1 to the CDT1-geminin hetero-trimer crystal structure (PBD ID 2WVR, both shown in cartoon with CDT1 in blue and geminin in green). The N-terminal binding surface of CDT1 and geminin is not occluded by the interactions between CDT1 and MCM2. **b** Side-by-side comparison of the elements from each structure. Figure made and alignments performed using Chimera X. **c** Sequence conservation of the geminin coiled-coil across human, mouse, bovine, *Xenopus,* and *Drosophila* metazoans. The conserved leucine zipper and regions of steric clash with MCM2 (residues 127-160 geminin chain A and 133-160 for chain B) are indicated. Alignment performed using Clustal Omega and ESPript. Red boxes with white characters denote strict identity, red characters denote similarity and blue frames indicate similarity across a group of residues. **d** AlphaFold model of the geminin coiled-coil (residues 96-160) highlights that the identical amino acids in part (**c**) (shown in red with side chains) are mostly at/near the interface of the coiled-coil.

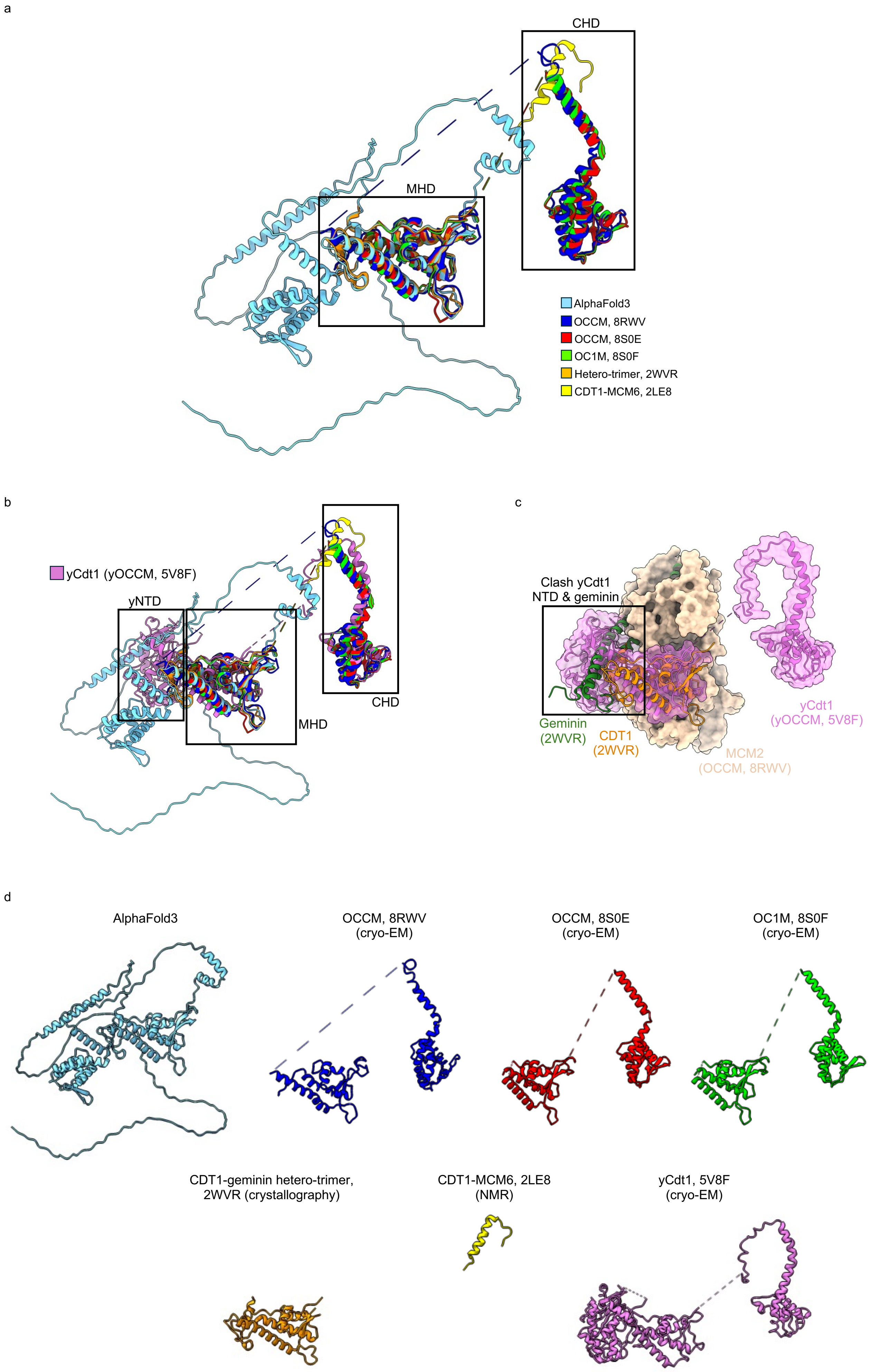
a
CHD
MHD
AlphaFold3
OCCM, 8RWV
OCCM, 8S0E
OC1M, 8S0F
Hetero-trimer, 2WVR
CDT1-MCM6, 2LE8
b
yCdt1 (yOCCM, 5V8F)
yNTD
CHD
MHD
c
Clash yCdt1
NTD & geminin
Geminin
(2WVR)
CDT1
(2WVR)
MCM2
(OCCM, 8RWV)
yCdt1
(yOCCM, 5V8F)
d
AlphaFold3
OCCM, 8RWV
(cryo-EM)
OCCM, 8S0E
(cryo-EM)
OC1M, 8S0F
(cryo-EM)
CDT1-geminin hetero-trimer,
2WVR (crystallography)
CDT1-MCM6, 2LE8
(NMR)
yCdt1, 5V8F
(cryo-EM)

Supplementary Figure 18: Comparing CDT1 structures

a Overlay of human CDT1 structures from the Protein Data Bank (PDB) and our AlphaFold3 model. Models were aligned to CDT1 of 8RWV, except 2LE2, which due to the short helix was aligned by MCM6. Whilst there is good alignment with the middle helical domain (MHD, residues 166-351) the AlphaFold3 model is not restrained by contacts with MCM, therefore the C-terminal helical domain (CHD, residues 352-546) does not align. The AlphaFold3 model also contains unstructured regions in the human CDT1 N-terminus (residues 1-165) that are unmodelled in the cryo-EM structures. **b** Part (**a**) overlaid with yCdt1 from the yeast OCCM structure (pink). The structures align well in the CHD and MHD, but the extra region of the yeast N-terminal domain (yNTD) is visible compared to the human cryo-EM structures. **c** Overlaying human MCM2 (from OCCM structure, PDB ID 8RWV) with yCdt1 and the CDT1-geminin crystal structure (PDB ID 2WVR, aligned to OCCM CDT1) reveals a clash between the NTD of yCdt1 and geminin, providing mechanistic insight into why geminin is not present in yeast. **d** CDT1 structures from parts (**a-c**) tiled for side-by-side comparison. PDB IDs for the structures and method of structural determination are displayed above each cartoon. Figure made and alignments performed using Chimera X.

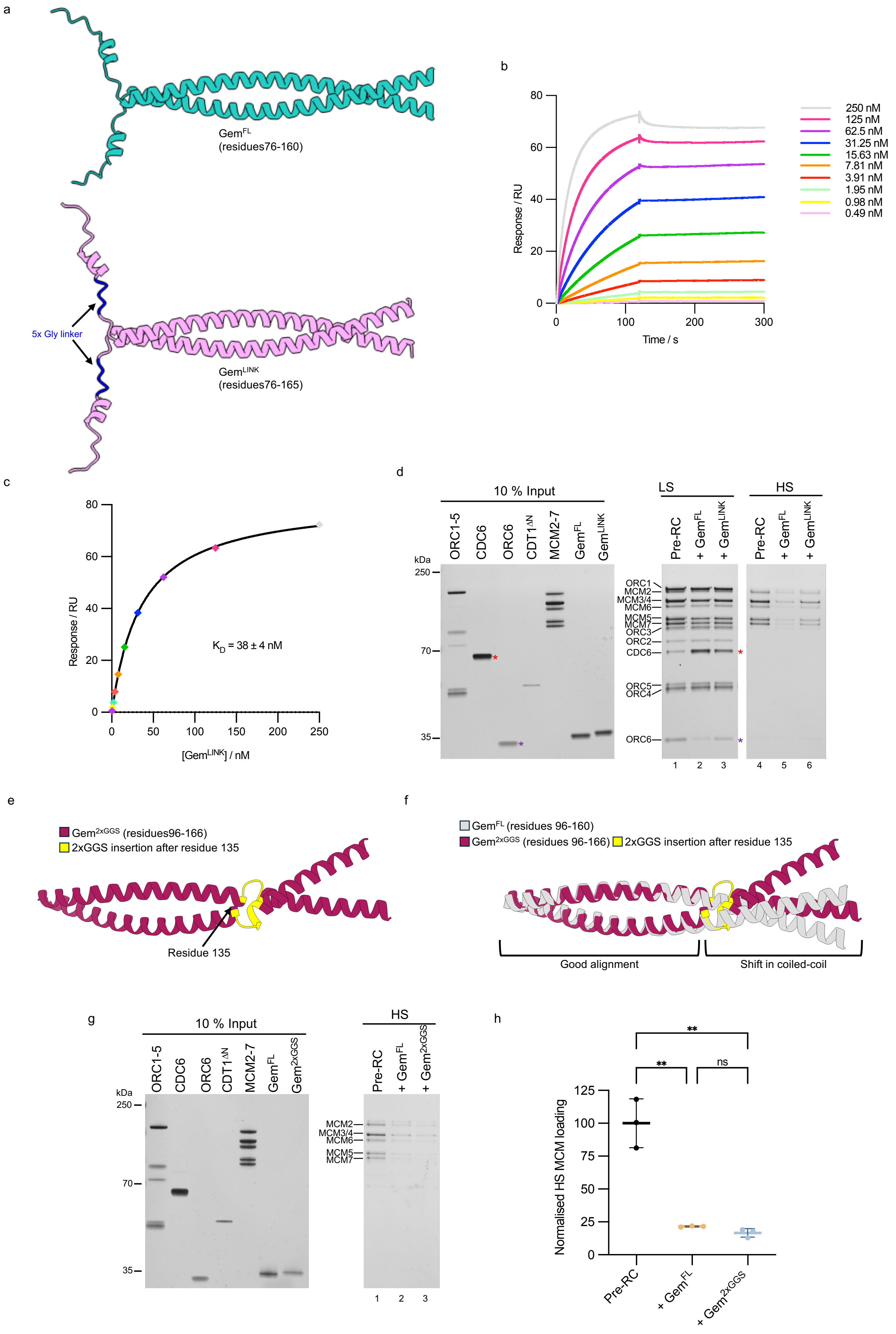
a
Gem^FL (residues76-160)
5x Gly linker
Gem^LINK (residues76-165)
b
Response / RU
Time / s
250 nM
125 nM
62.5 nM
31.25 nM
15.63 nM
7.81 nM
3.91 nM
1.95 nM
0.98 nM
0.49 nM
c
Response / RU
K_D = 38 ± 4 nM
[Gem^LINK] / nM
d
10 % Input
ORC1-5
CDC6
ORC6
CDT1^ΔN
MCM2-7
Gem^FL
Gem^LINK
LS
HS
Pre-RC
+ Gem^FL
+ Gem^LINK
kDa
250
70
35
ORC1
MCM2
MCM3/4
MCM6
MCM5
MCM7
ORC3
ORC2
CDC6
ORC5
ORC4
ORC6
1 2 3 4 5 6
e
Gem^2xGGS (residues96-166)
2xGGS insertion after residue 135
Residue 135
f
Gem^FL (residues 96-160)
Gem^2xGGS (residues 96-166)
2xGGS insertion after residue 135
Good alignment
Shift in coiled-coil
g
10 % Input
ORC1-5
CDC6
ORC6
CDT1^ΔN
MCM2-7
Gem^FL
Gem^2xGGS
HS
Pre-RC
+ Gem^FL
+ Gem^2xGGS
kDa
250
70
35
MCM2
MCM3/4
MCM6
MCM5
MCM7
1 2 3
h
**
**
ns
Normalised HS MCM loading
Pre-RC
+ Gem^FL
+ Gem^2xGGS

Supplementary Figure 19: Investigating geminin mutants in DNA replication licensing.

a AlphaFold prediction showing residues 76-160 of a geminin dimer (Gem^{FL}, teal) and residues 76-165 of the Gem^{LINK} (pink). The position of the 5x glycine linker between I90 and K96 (numbering adjusted for insertion of glycines) is shown in blue. Figure made using Chimera X. **b** Representative multi-cycle SPR sensorgram and corresponding affinity modelling (**c**) showing the binding of Gem^{LINK} to $CDT1^{\Delta N}$. **d** LS and HS pre-RC reactions of Gem^{FL} and Gem^{LINK}. Gem^{LINK} is less potent than Gem^{FL} in inhibiting stable MCM2-7 recruitment. * red highlights the stabilisation of CDC6 and * purple denotes the inhibited ORC6 recruitment observed with Gem^{LINK}. **e** AlphaFold model of the geminin coiled-coil with a 2xGGS insertion after residue 135 (Gem^{2xGGS}, inserted residues highlighted in yellow). **f** Overlay of the Gem^{2xGGS} coiled-coil (burgundy, with insertion in yellow) with Gem^{FL} (grey). Prior to the 2xGGS insertion, there is good alignment of the coiled-coil. Insertion of 2xGGS shifts the positions of the coiled-coil. **g** Pre-RC assay demonstrating that Gem^{2xGGS} can block loading of the salt-stable MCM2-7 double hexamer. **h** Quantification of the mean ±SD of MCM2-7 band intensity in part (**g**) relative to the control pre-RC reaction. Data was compared to the control pre-RC reaction using one-way ANOVA and Tukey's multiple comparison test. **p= from bottom to top; 0.0025, 0.0020. Representative of three biological repeats. Source data are provided as a Source Data file.

a

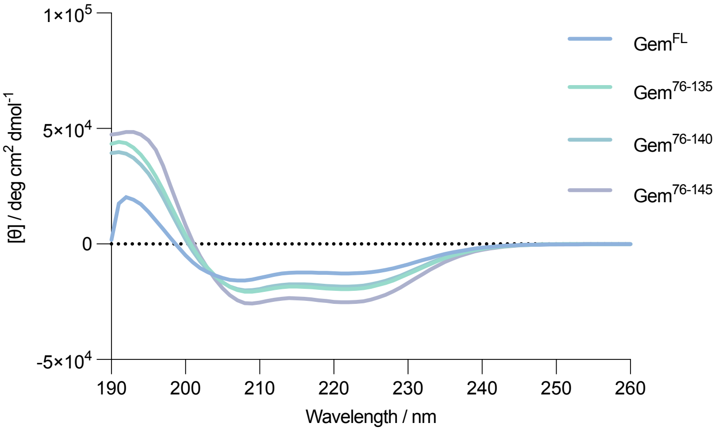

b

Peptide	Helicity / %	Oligomerisation state
Gem^{76-145}	70	2.10 ± 0.04
Gem^{76-140}	54	2.04 ± 0.05
Gem^{76-135}	57	2.02 ± 0.06

Supplementary Figure 20: Effect of C-terminal truncation of Gem^{76-145} on peptide helicity and oligomerisation

a CD spectra of ~50 µM of peptide dissolved in 10 mM phosphate buffer at pH 7.4. **b** Helicities of peptides, as determined using the K2D3 webserver[6]. SEC-MALS was used to determine oligomerisation states. Data displayed as the mean and SE of 3 independent SEC-MALS experiments. Source data are provided as a Source Data file.

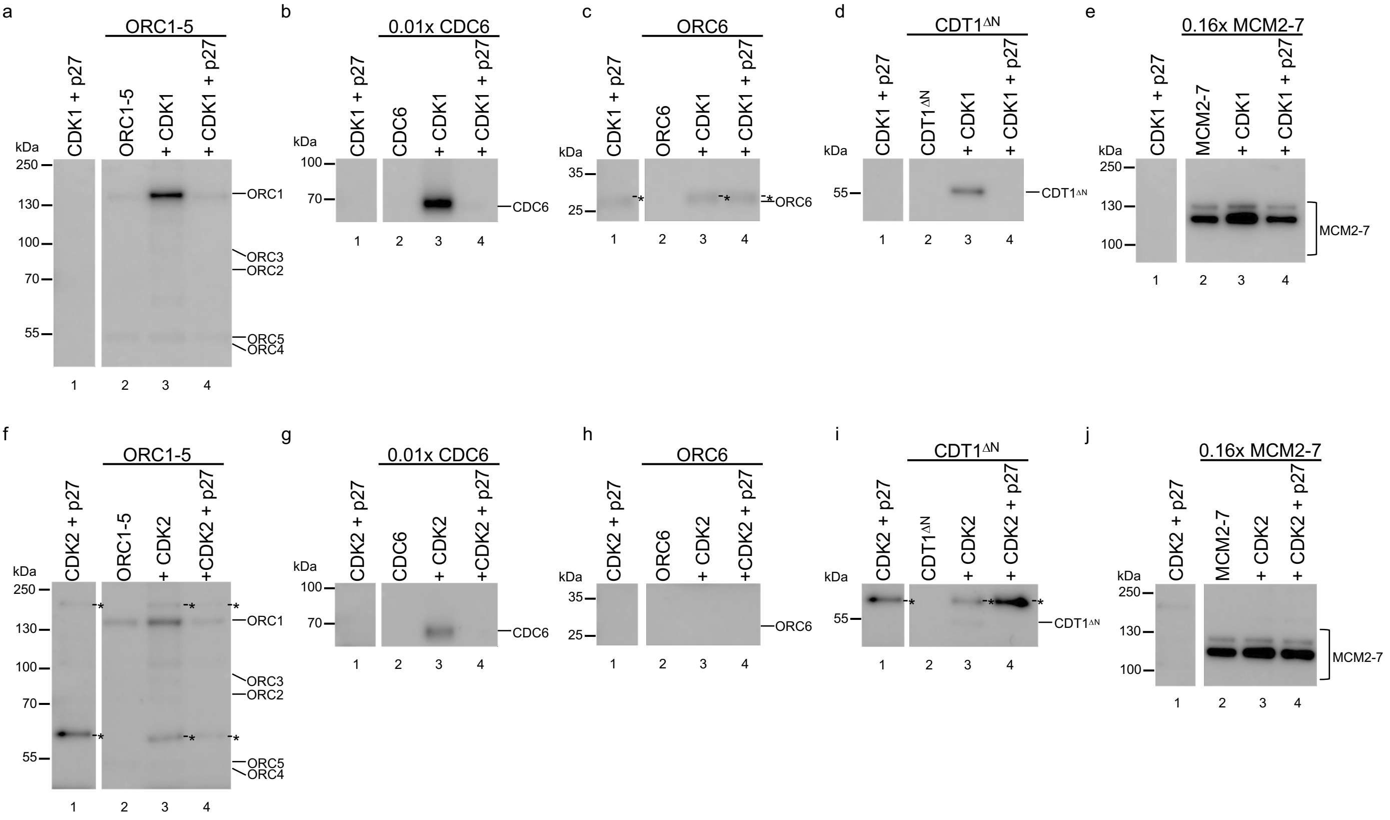

Supplementary Figure 21: CDK-phosphorylation of pre-RC proteins

Pre-RC proteins were phosphorylated using either CDK1-cyclin A2 (**a-e,** CDK1) or CDK2-cyclin A (**f-j**, CDK2) and subjected to blotting with an anti-CDK substrate motif [(K/H)pSP] antibody. Lanes 1: CDK + p27 input control. Non-specific bands from input are denoted by ¯*. Lanes 2: apo-protein. Lanes 3: pre-RC protein + CDK. Lanes 4: Pre-incubation of CDK with inhibitory p27 for 5 minutes prior to addition of pre-RC protein. For CDC6 (b and g, 0.01x) and MCM2-7 (e and j, 0.16x) the relative amount of the reaction loaded compared to the other panels was reduced due to high signal. Representative of three biological repeats. Source data are provided as a Source Data file.

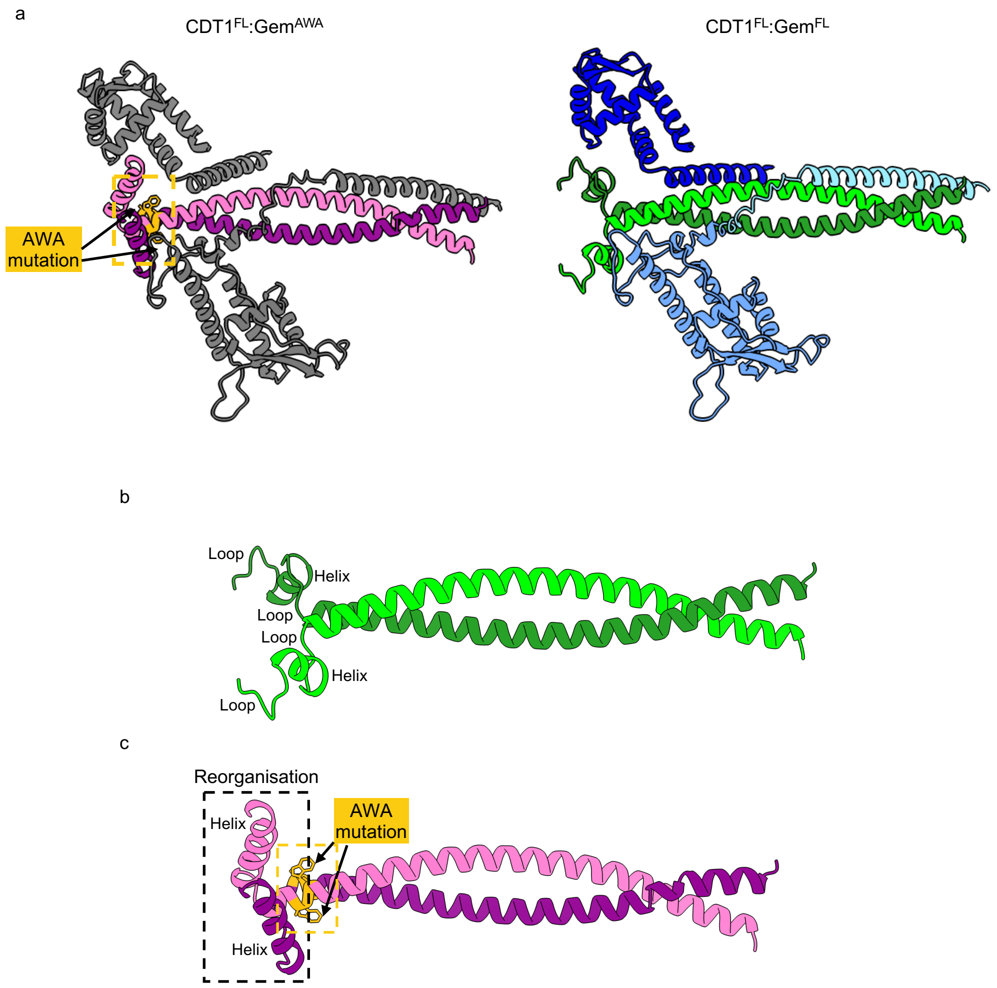

Supplementary Figure 22: The geminin^{AWA} mutant perturbs the loop, helix, loop

a AlphaFold models of geminin and CDT1. The overall architecture of the geminin^{AWA} mutant[8]; left geminin in pink/purple with mutated residues in orange) in complex with CDT1 (grey) is highly similar to that of wild-type geminin in complex with CDT1 (Left with geminin in green and CDT1 in blue). **b** The AlphaFold model of wild-type geminin has a loop, helix, loop motif prior to the coiled coil. **c** The AlphaFold model of the geminin^{AWA} mutant suggests that the mutation perturbs the loop, helix, loop motif, reorganising this region to a helix (black box). The change of this motif may explain why the mutant is permissive for helicase loading.

a

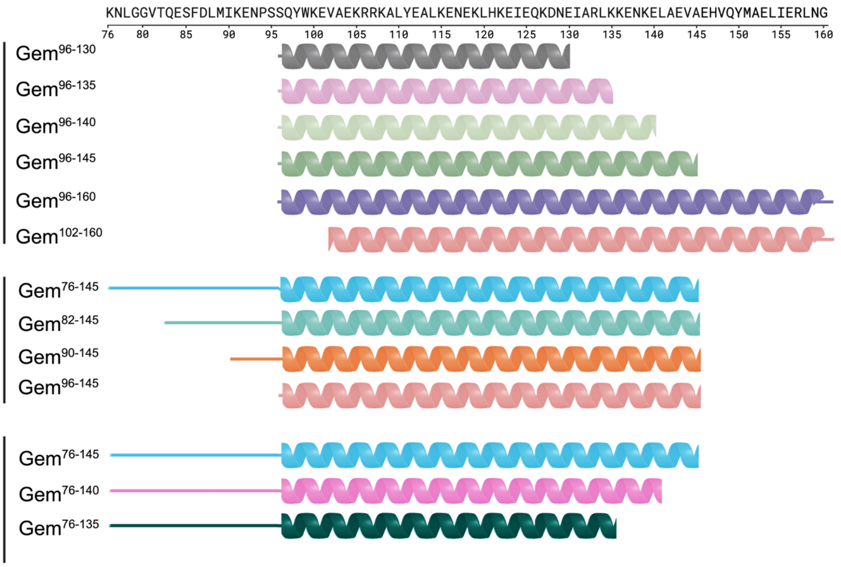

b

Peptide	Yield / mg	Yield / %	M_W (calc) / g mol^{-1}	M_W (LC-MS) / g mol^{-1}
Gem$^{96\text{-}130}$	19.0	17	4402.2934	4402.31
Gem$^{96\text{-}135}$	5.0	4	4983.6947	4983.71
Gem$^{96\text{-}140}$	17.2	12	5613.0155	5613.04
Gem$^{96\text{-}145}$	12.0	8	6096.2848	6096.30
Gem$^{96\text{-}160}$	18.0	9	7880.1596	7880.17
Gem$^{102\text{-}160}$	5.6	3	7058.7887	7058.80
Gem$^{76\text{-}145}$	10.5	5	8286.3734	8286.40
Gem$^{82\text{-}145}$	18.6	10	7717.0375	7717.07
Gem$^{90\text{-}145}$	25.5	15	6764.6342	6764.66
Gem$^{76\text{-}140}$	6.2	3	7802.0828	7802.13
Gem$^{76\text{-}135}$	5.1	3	7173.7833	7173.81
Gem^{I131A}	8.4	4	8243.3238	8243.35
Gem^{L134A}	10.7	5	8243.3238	8243.35
Gem^{I131A,L134A}	8.5	4	8201.2769	8201.30

Supplementary Figure 23: Summary of geminin-mimetic peptides used in this study

a Graphical summary of peptides with amino acid sequence and residue numbers shown above. Created in BioRender. Faull, S. (2025) https://BioRender.com/o3ugqw3. **b** Yields and M_W of peptides synthesised using SPPS. M_W were determined using the high-resolution LC-MS method and compared to M_W calculated for the most abundant isotope.

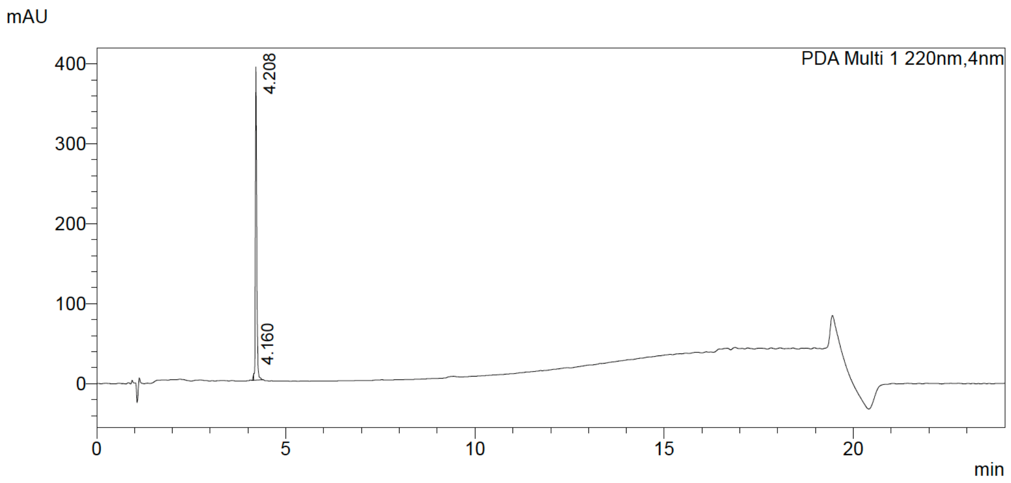

Supplementary Figure 24a: HPLC trace of the pure Gem$^{96\text{-}130}$ peptide

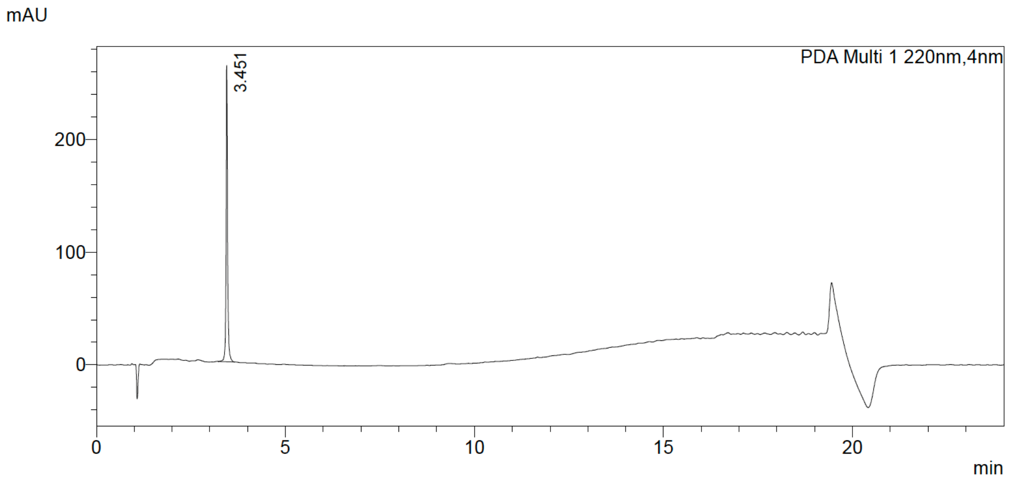

Supplementary Figure 24b: HPLC trace of the pure Gem$^{96\text{-}135}$ peptide

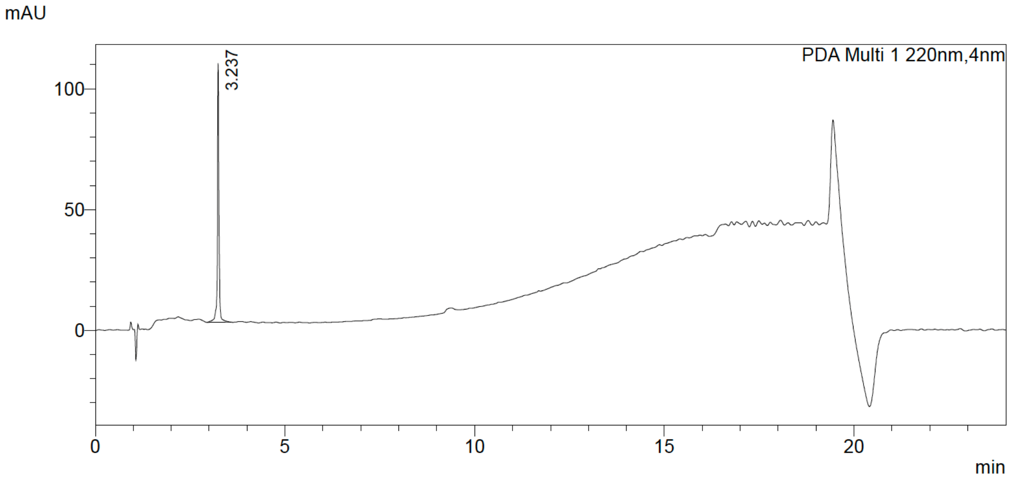

Supplementary Figure 24c: HPLC trace of the pure Gem$^{96\text{-}140}$ peptide

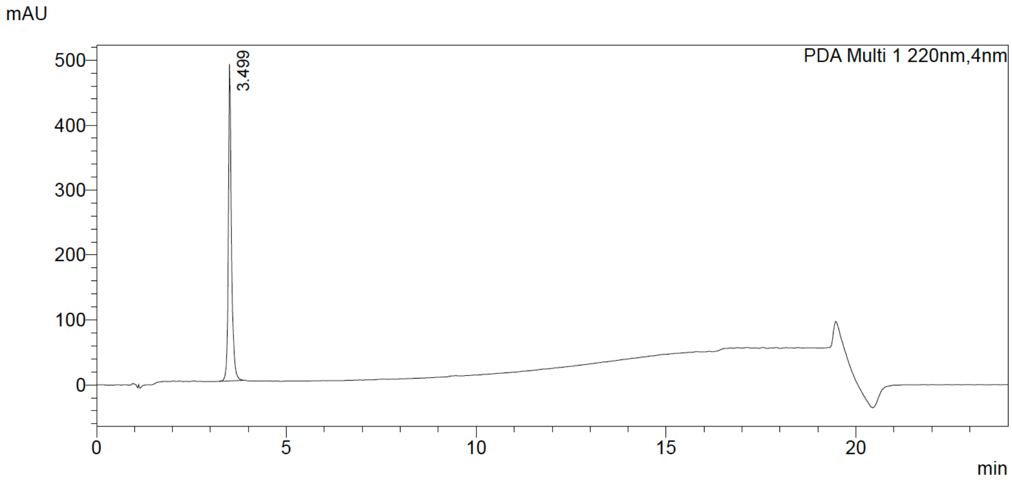

Supplementary Figure 24d: HPLC trace of the pure Gem$^{96\text{-}145}$ peptide

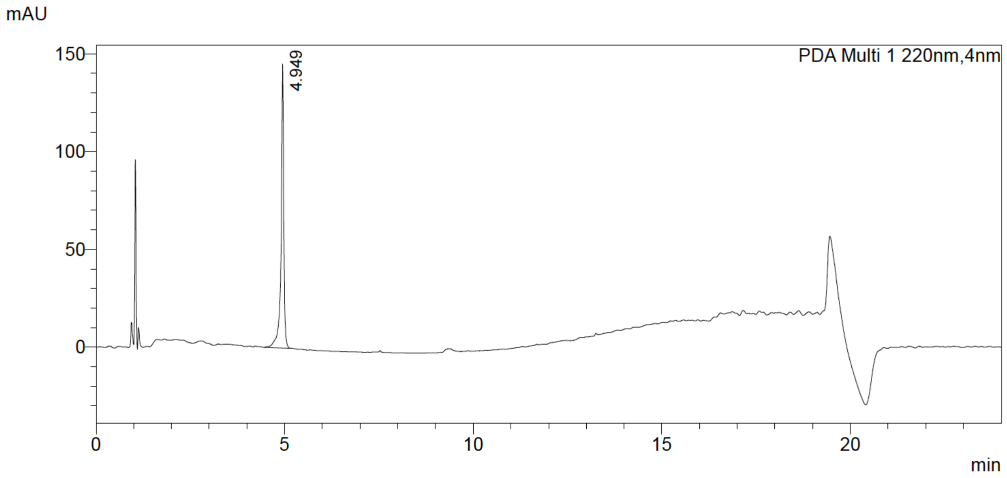

Supplementary Figure 24e: HPLC trace of the pure Gem$^{96\text{-}160}$ peptide

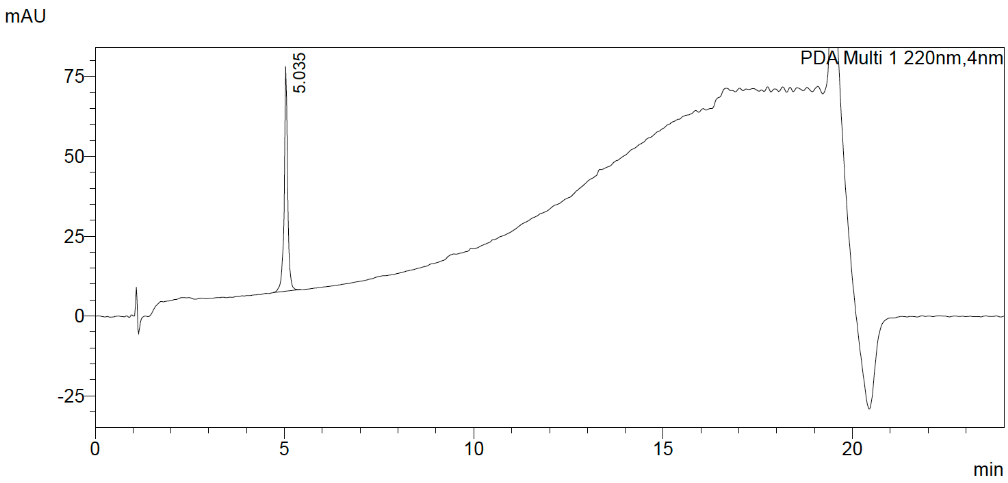

Supplementary Figure 24f: HPLC trace of the pure Gem$^{102\text{-}160}$ peptide

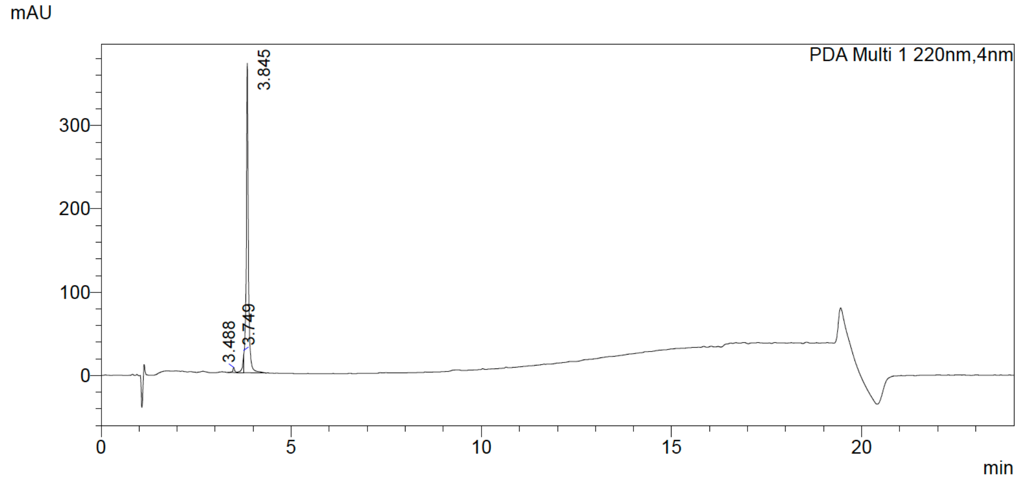

Supplementary Figure 24g: HPLC trace of the pure Gem$^{76\text{-}145}$ peptide

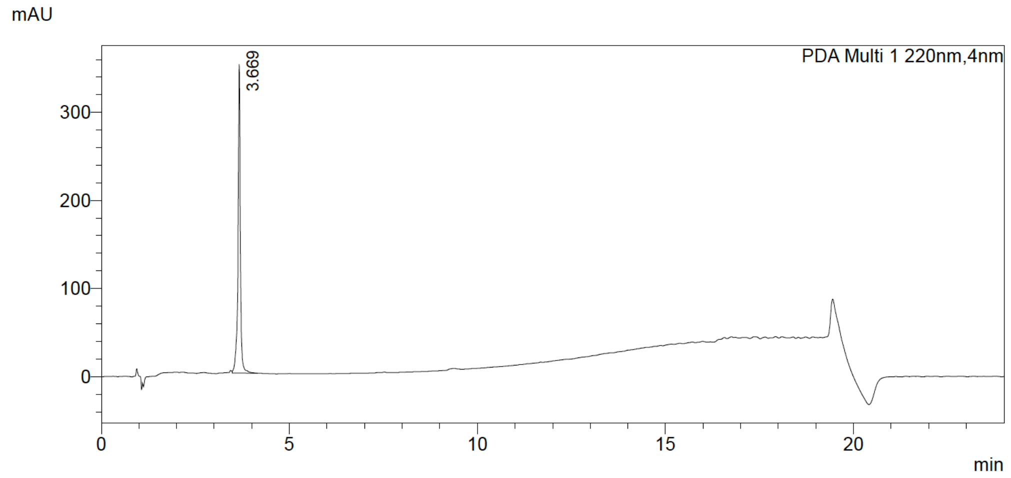

Supplementary Figure 24h: HPLC trace of the pure Gem$^{82\text{-}145}$ peptide

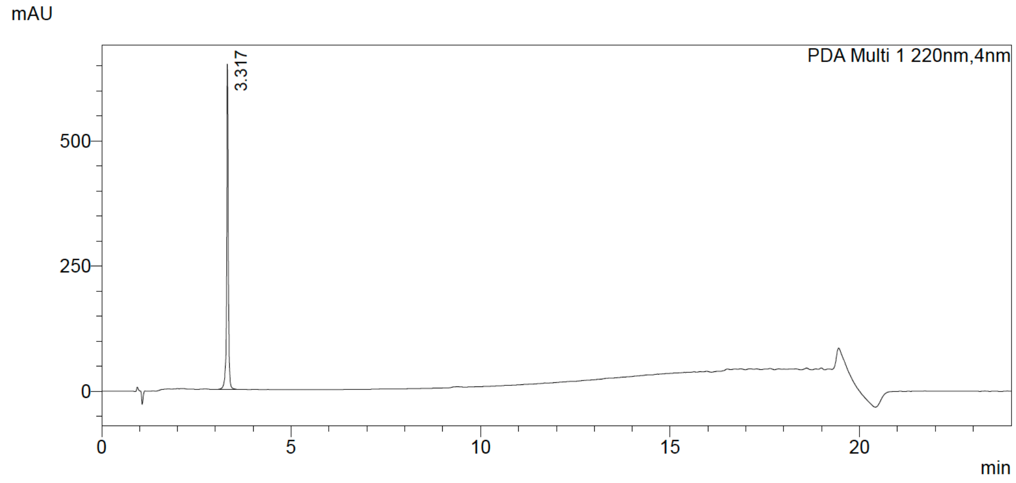

Supplementary Figure 24i: HPLC trace of the pure Gem$^{90\text{-}145}$ peptide

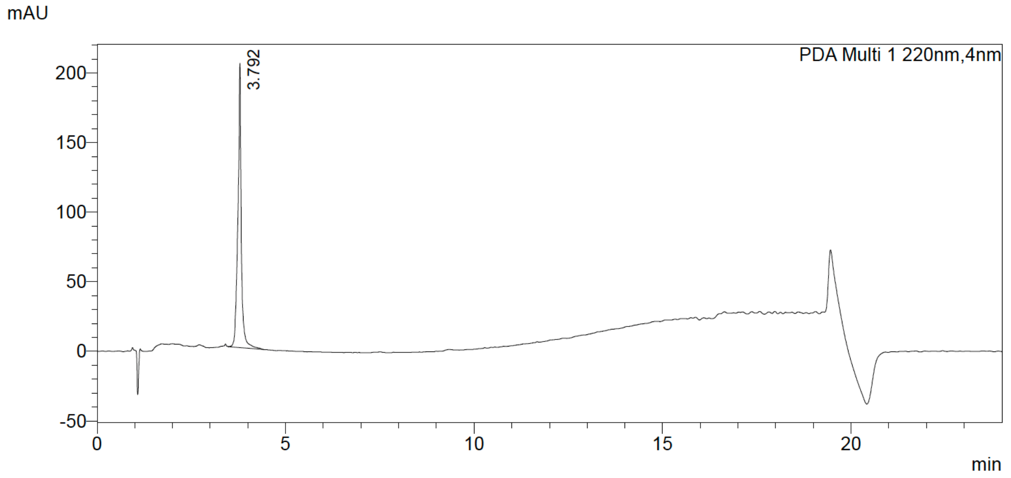

Supplementary Figure 24j: HPLC trace of the pure Gem$^{76\text{-}140}$ peptide

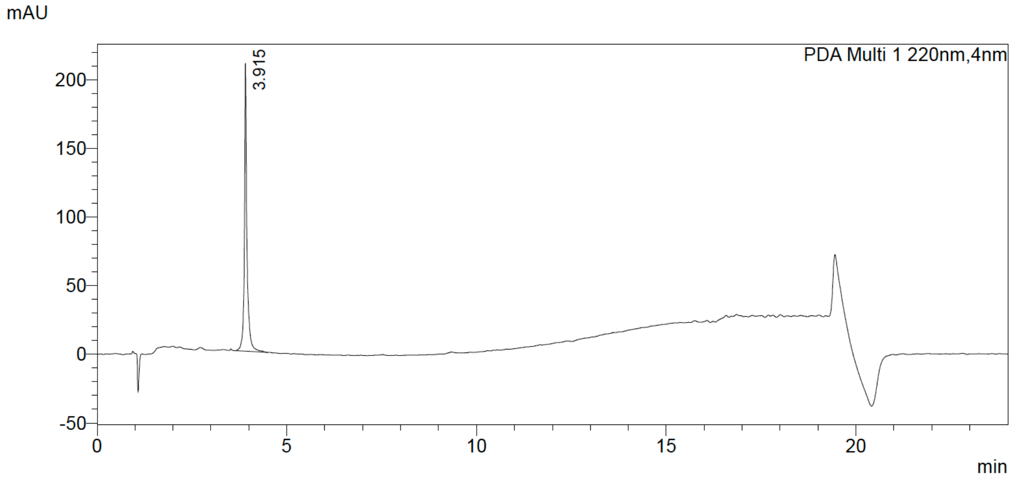

Supplementary Figure 24k: HPLC trace of the pure Gem$^{76\text{-}135}$ peptide

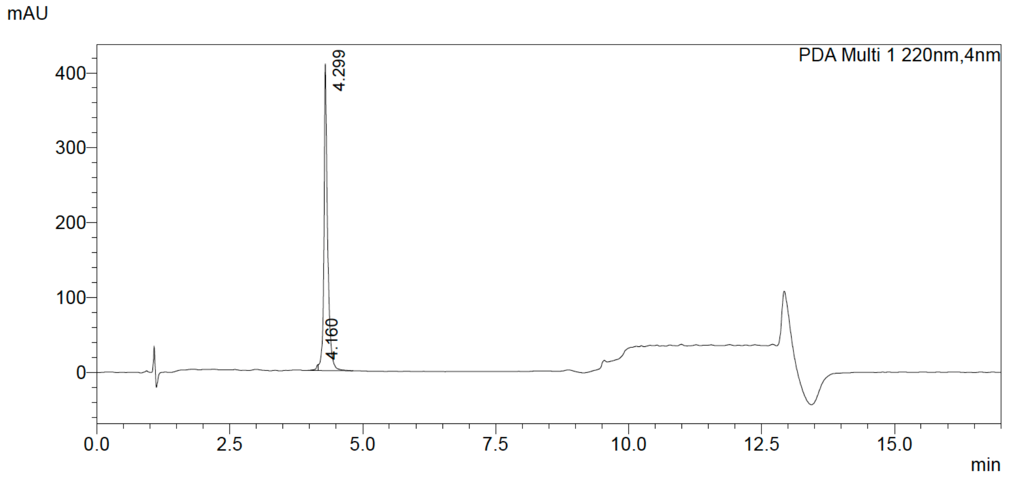

Supplementary Figure 24l: HPLC trace of the pure Gem^{I131A} peptide

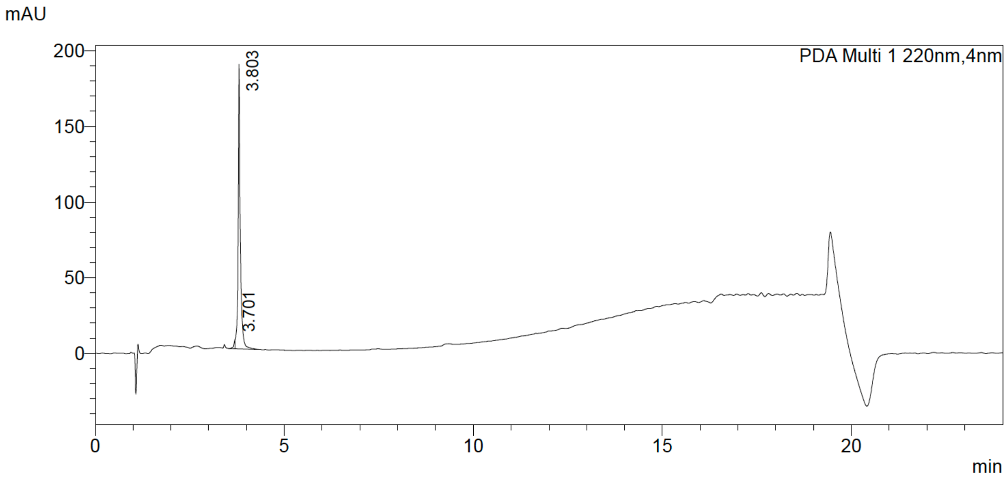

Supplementary Figure 24m: HPLC trace of the pure Gem^{L134A} peptide

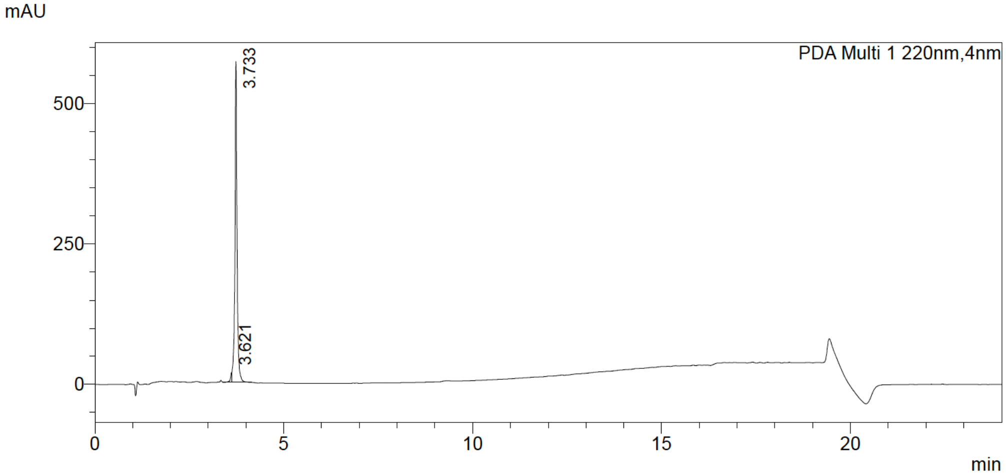

Supplementary Figure 24n: HPLC trace of the pure Gem^{I131A,L134A} peptide

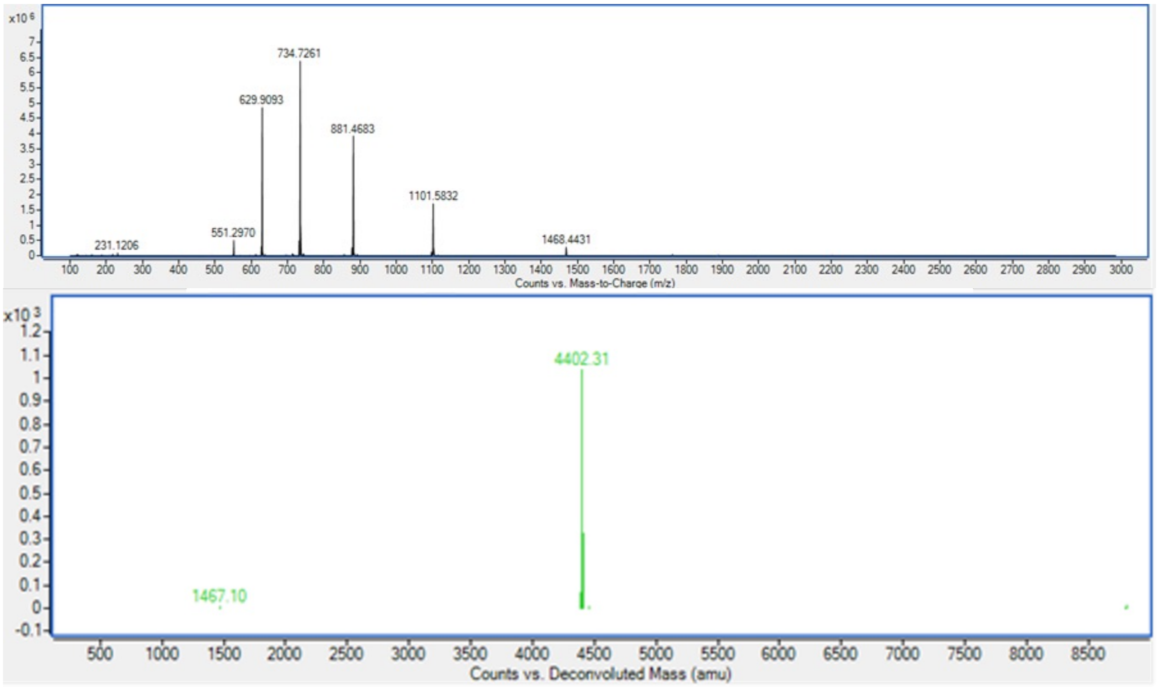

Supplementary Figure 25a: Raw (top) and deconvoluted (bottom) mass spectra of pure Gem$^{96\text{-}130}$

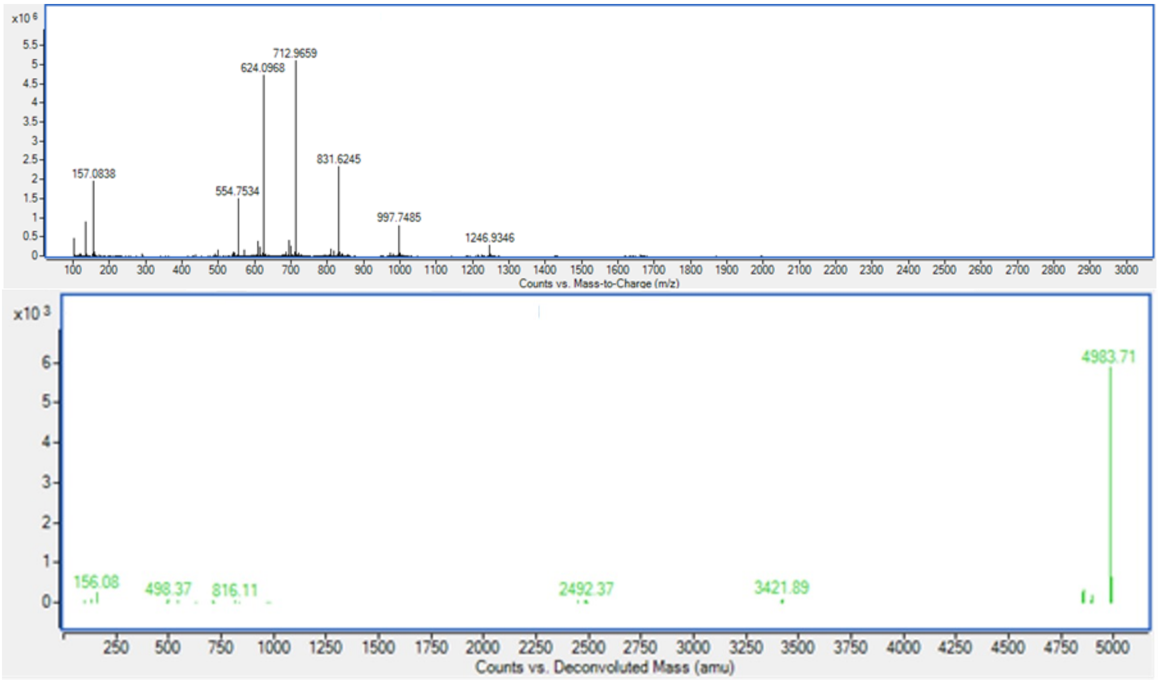

Supplementary Figure 25b: Raw (top) and deconvoluted (bottom) mass spectra of pure Gem$^{96\text{-}135}$

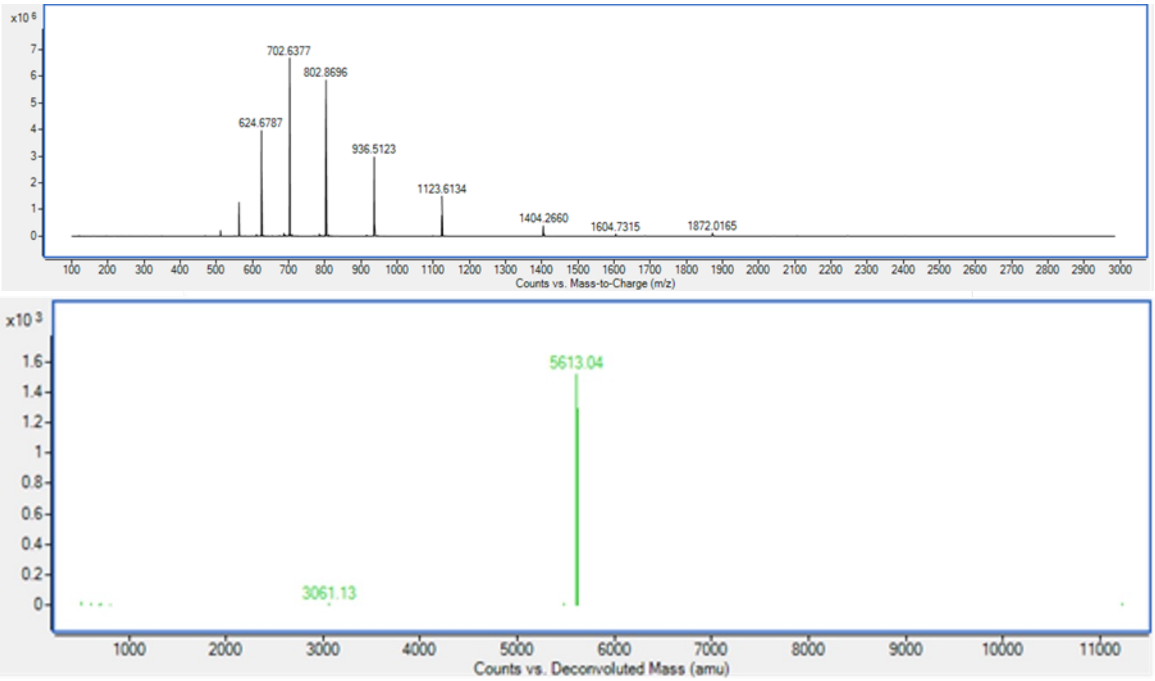

Supplementary Figure 25c: Raw (top) and deconvoluted (bottom) mass spectra of pure Gem$^{96\text{-}140}$

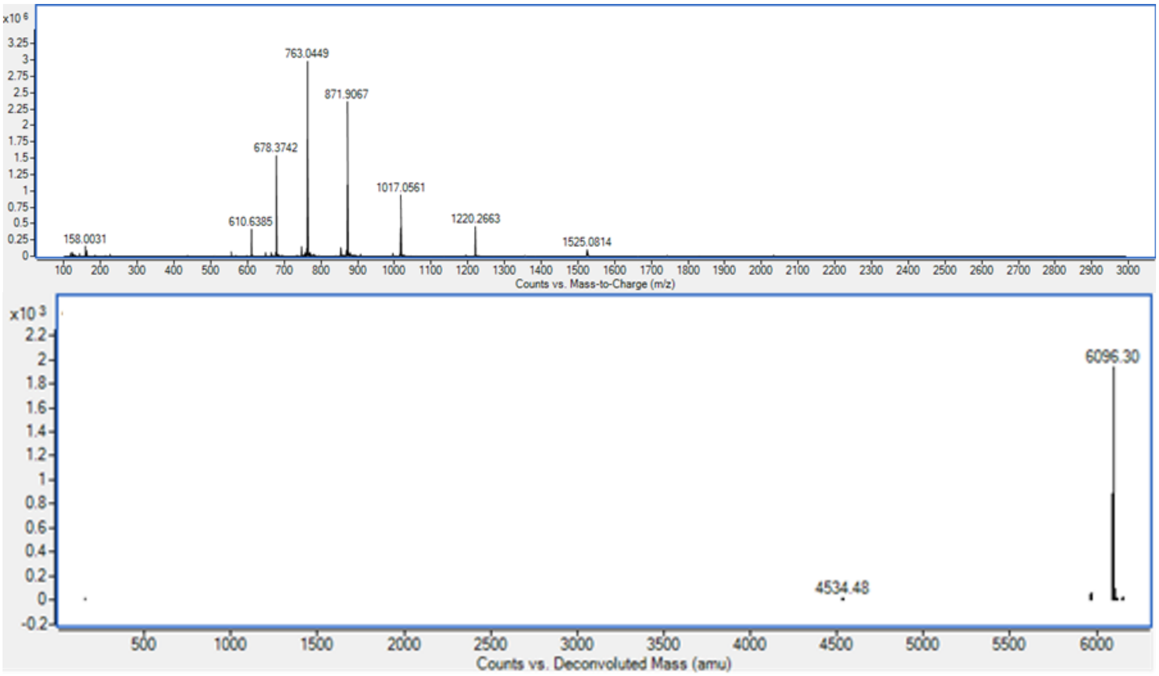

Supplementary Figure 25d: Raw (top) and deconvoluted (bottom) mass spectra of pure Gem$^{96\text{-}145}$

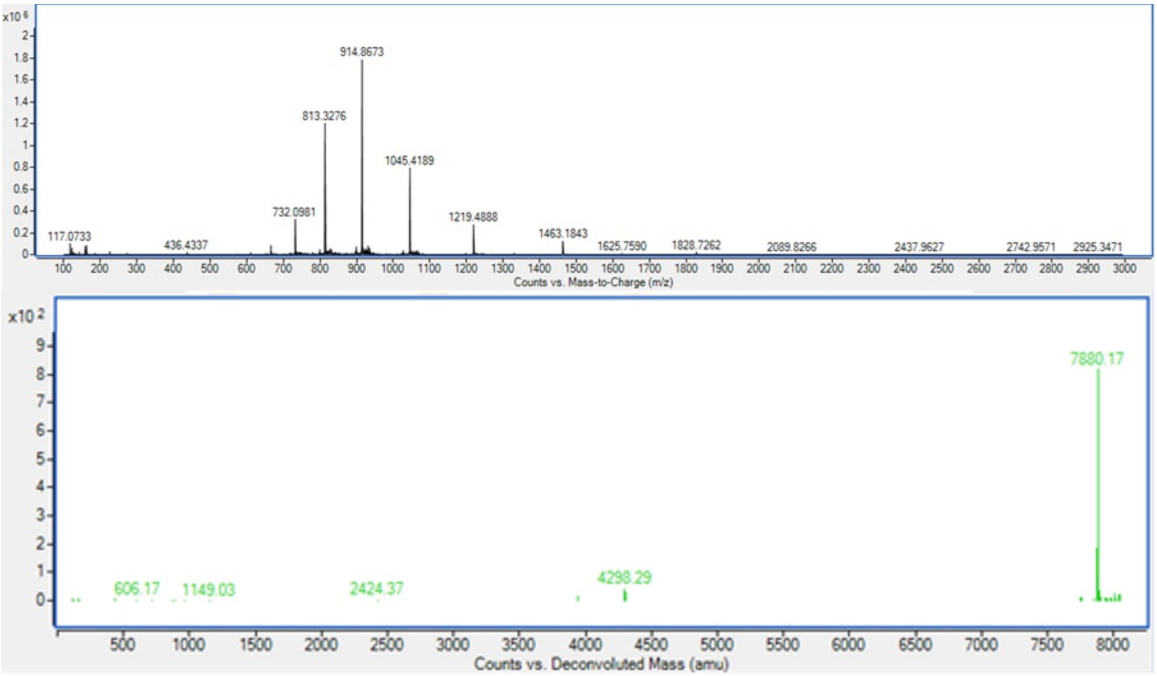

Supplementary Figure 25e: Raw (top) and deconvoluted (bottom) mass spectra of pure Gem$^{96-160}$

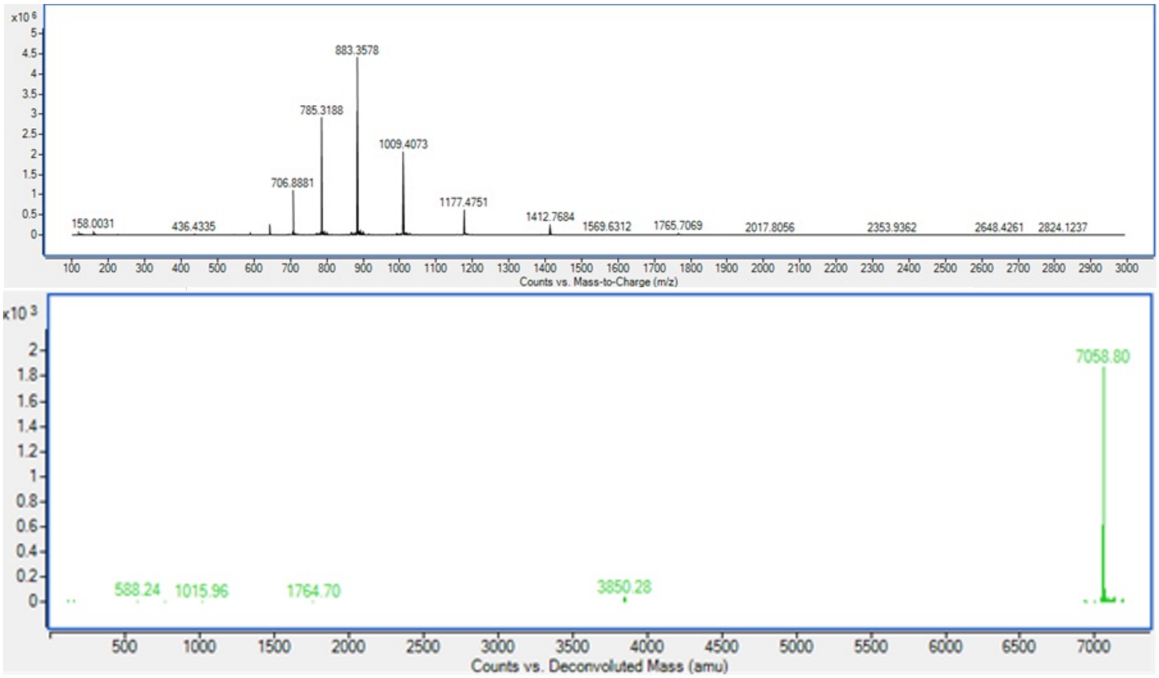

Supplementary Figure 25f: Raw (top) and deconvoluted (bottom) mass spectra of pure Gem$^{102-160}$

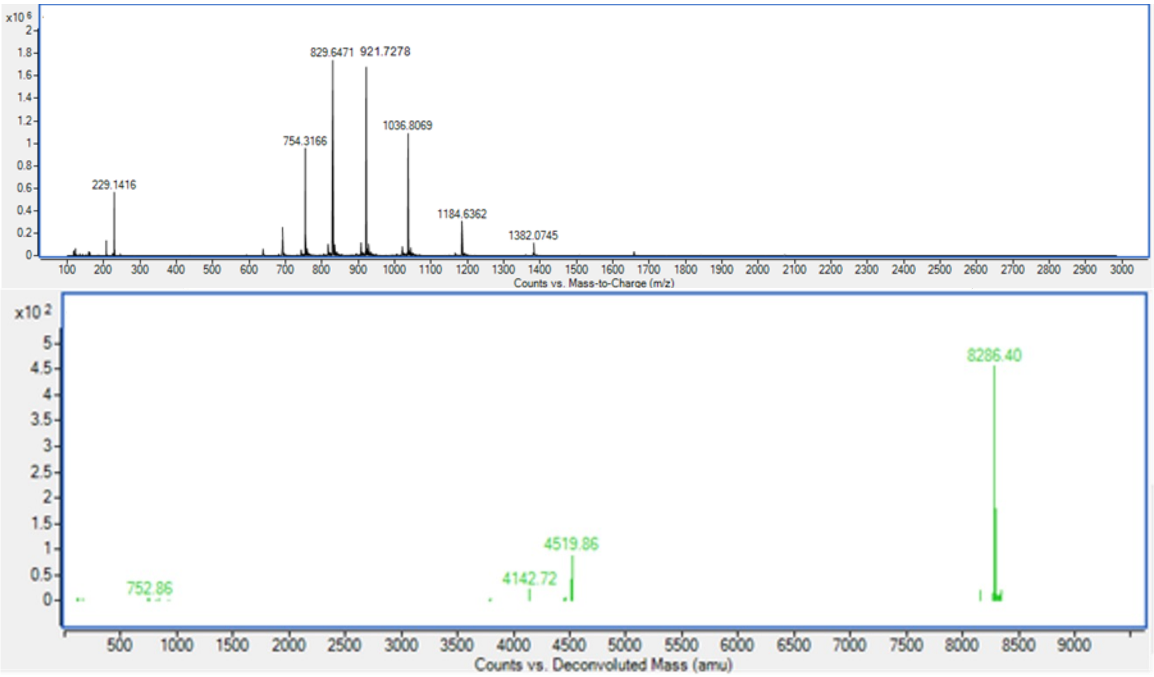

Supplementary Figure 25g: Raw (top) and deconvoluted (bottom) mass spectra of pure Gem$^{76\text{-}145}$

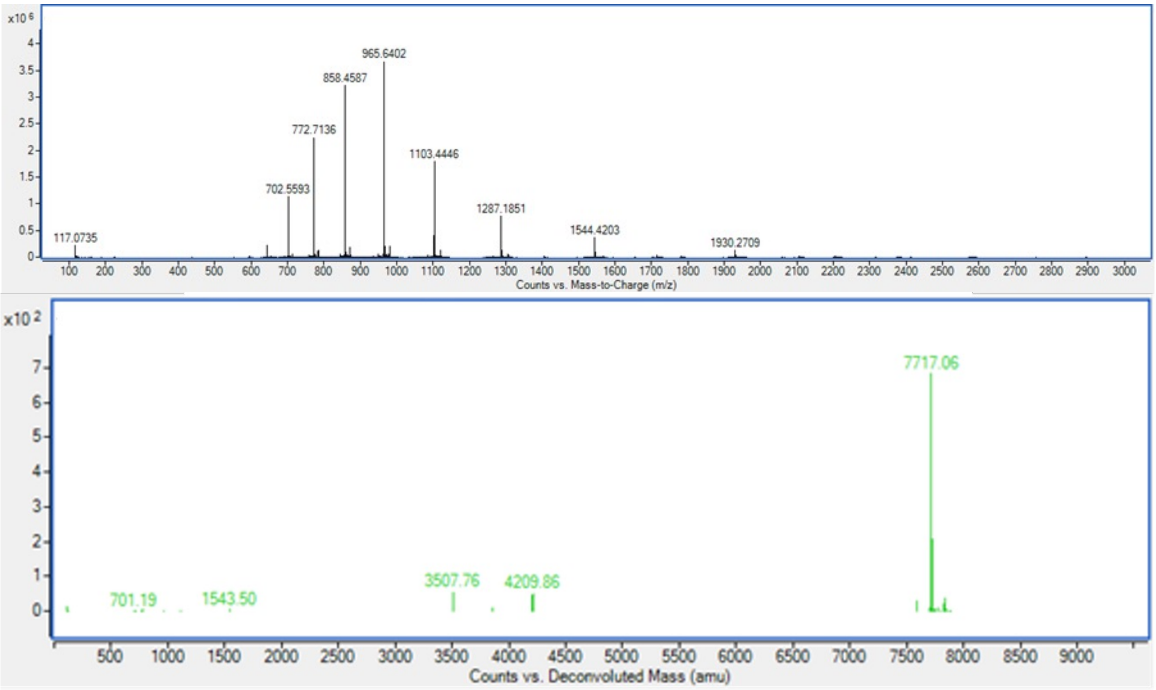

Supplementary Figure 25h: Raw (top) and deconvoluted (bottom) mass spectra of pure Gem$^{82\text{-}145}$

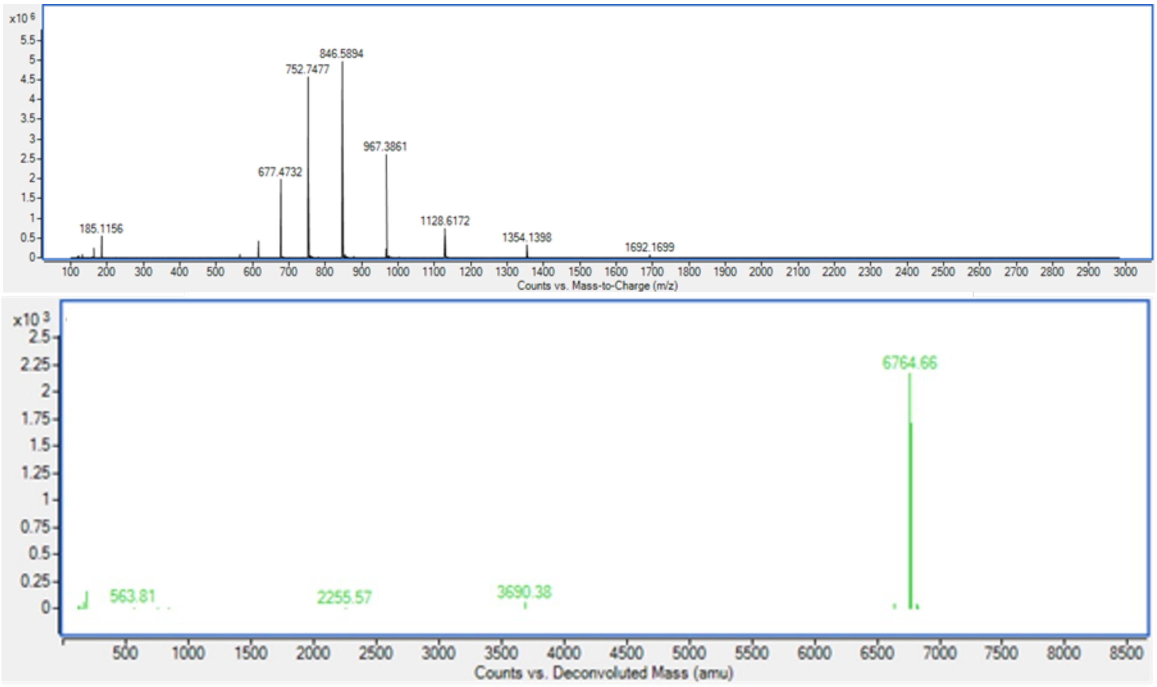

Supplementary Figure 25i: Raw (top) and deconvoluted (bottom) mass spectra of pure Gem$^{90\text{-}145}$

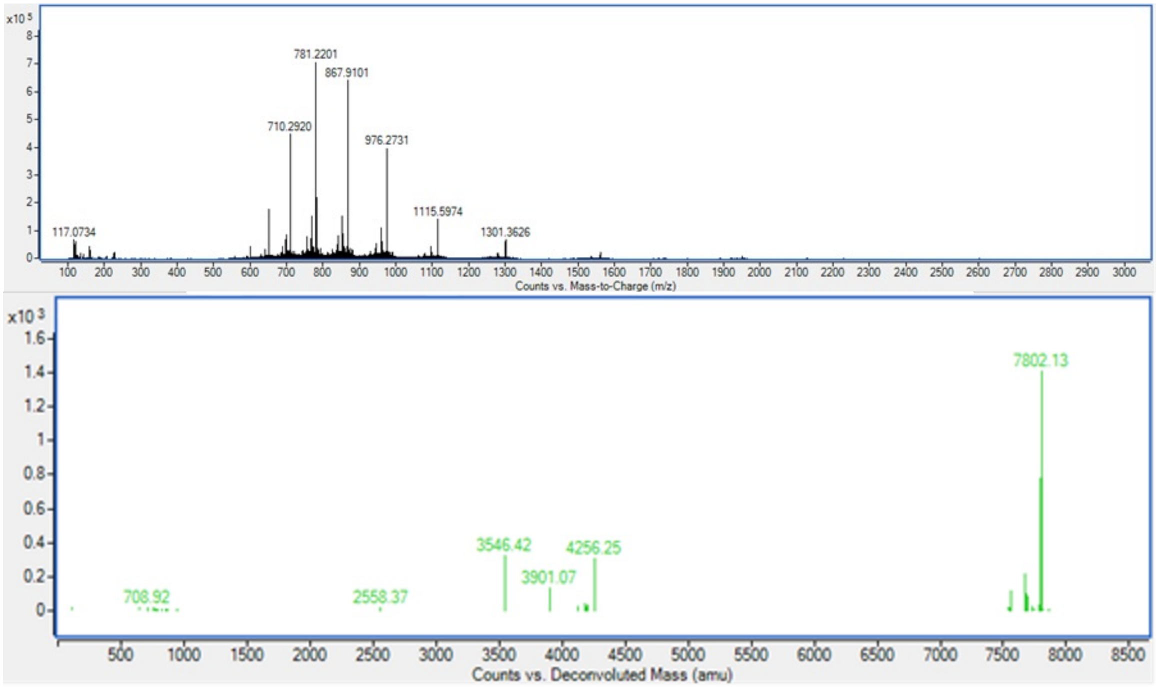

Supplementary Figure 25j: De Raw (top) and deconvoluted (bottom) mass spectra of pure Gem$^{76\text{-}140}$

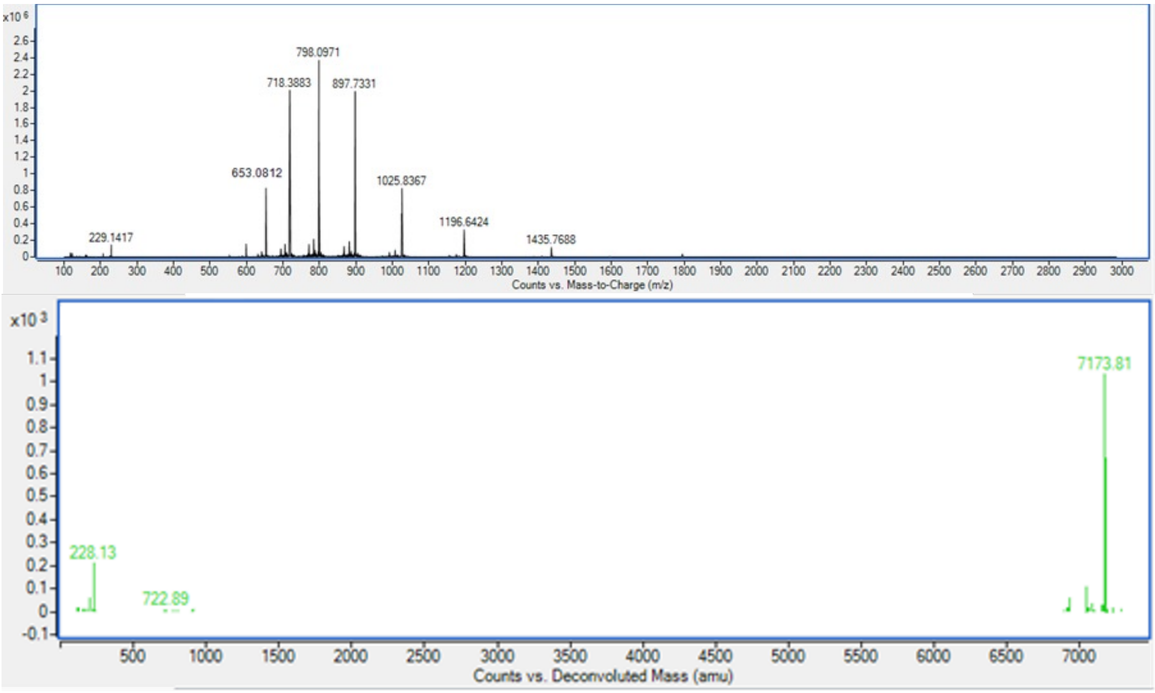

Supplementary Figure 25k: Raw (top) and deconvoluted (bottom) mass spectra of pure Gem$^{76\text{-}135}$

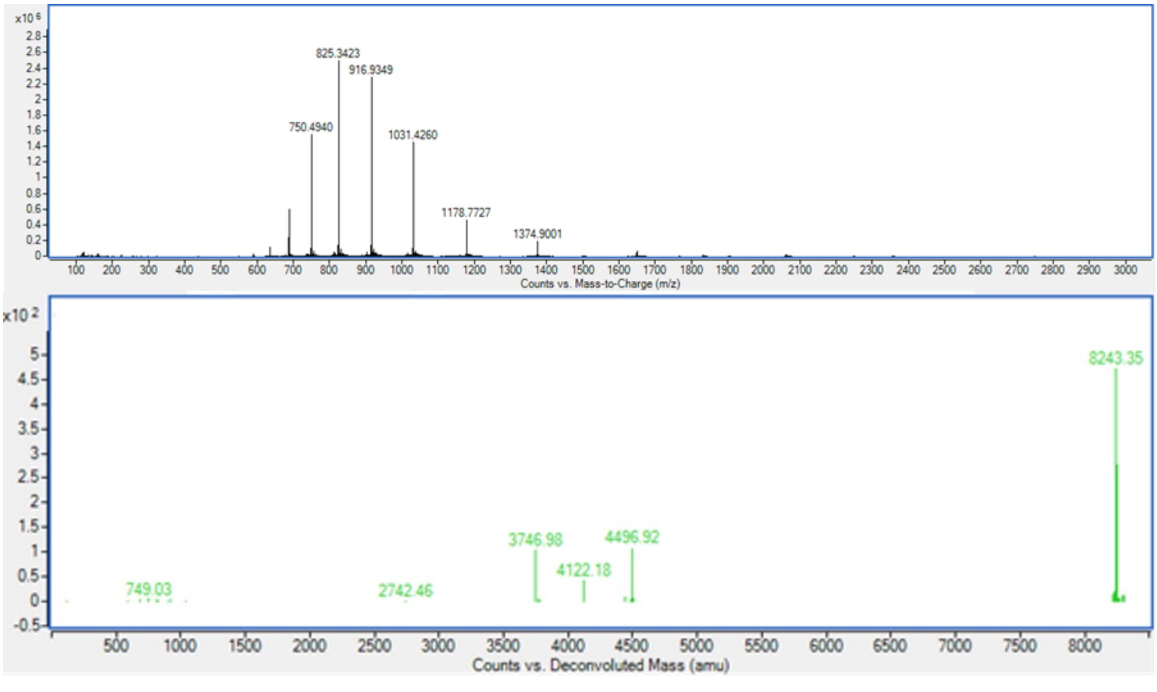

Supplementary Figure 25l: Raw (top) and deconvoluted (bottom) mass spectra of pure Gem^{I131A}

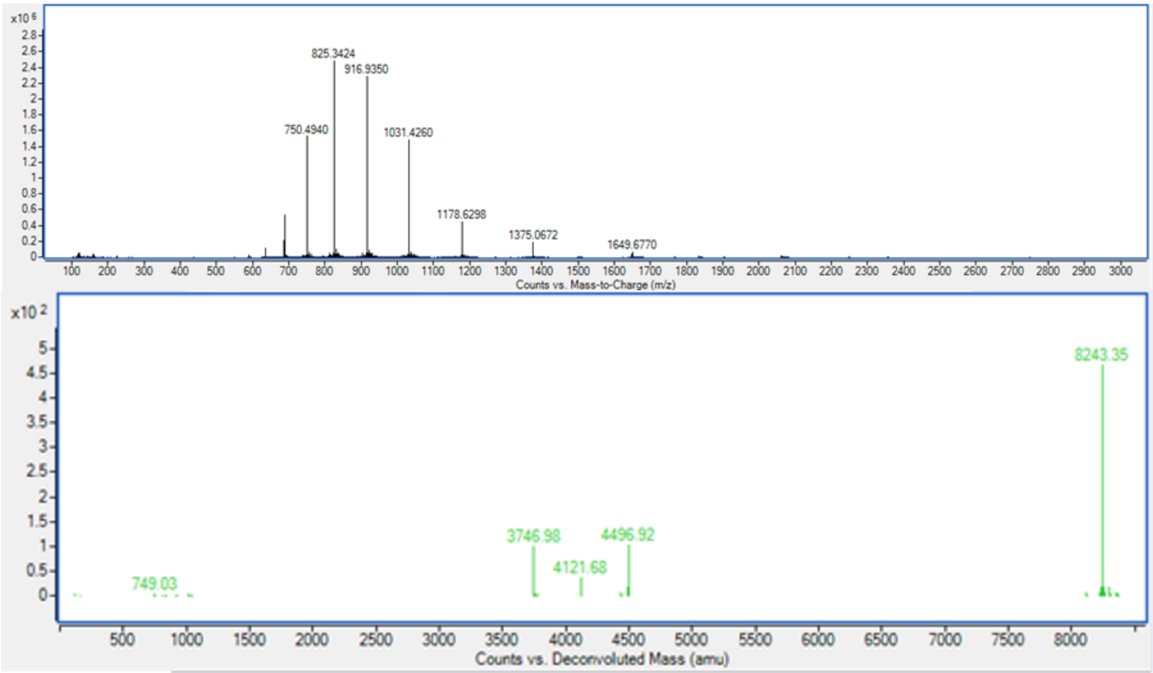

Supplementary Figure 25m: Raw (top) and deconvoluted (bottom) mass spectra of pure Gem^{L134A}

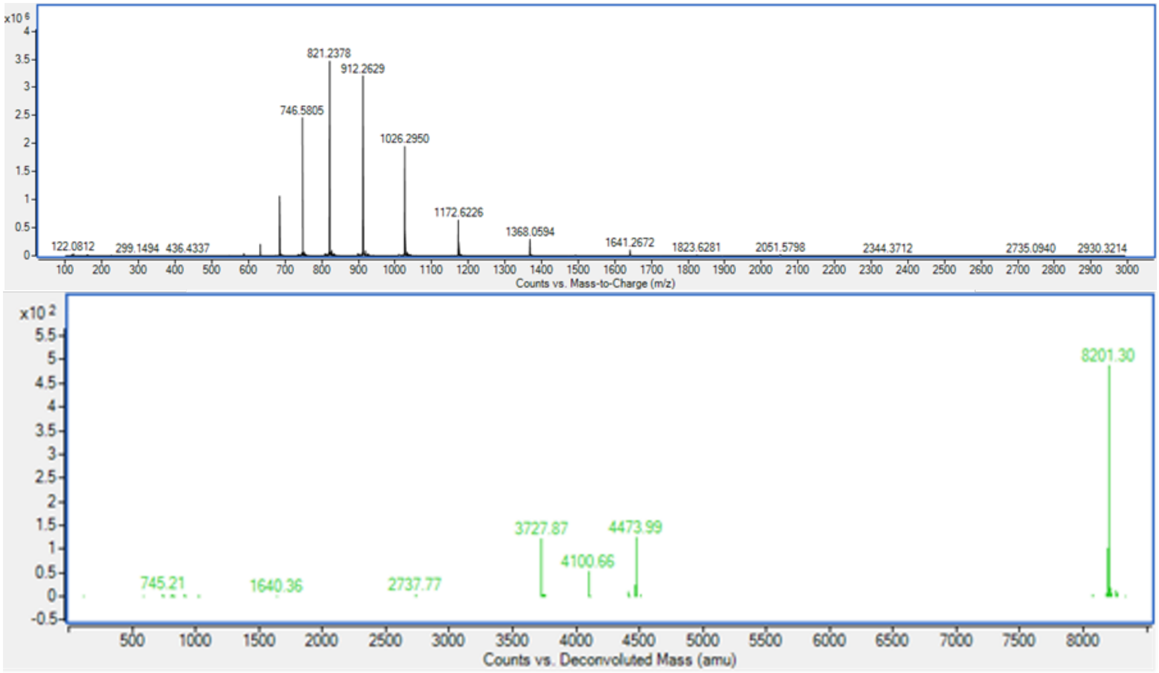

Supplementary Figure 25n: Raw (top) and deconvoluted (bottom) mass spectra of pure Gem$^{I131A,\ L134A}$

SUPPLEMENTARY REFERENCES

1. Yang, R., Hunker, O., Wise, M. & Bleichert, F. Multiple mechanisms for licensing human replication origins. *Nature* **636**, 488-498 (2024).

2. Weissmann, F. et al. MCM double hexamer loading visualized with human proteins. *Nature* **636**, 499-508 (2024).

3. Kulartz, M. & Knippers, R. The replicative regulator protein geminin on chromatin in the HeLa cell cycle. *J Biol Chem* 279, 41686-94 (2004).

4. Hochegger, H., Takeda, S. & Hunt, T. Cyclin-dependent kinases and cell-cycle transitions: does one fit all? *Nat Rev Mol Cell Biol* **9**, 910–916 (2008).

5. Yariv, B. et al. Using evolutionary data to make sense of macromolecules with a "face-lifted" ConSurf. Protein Sci 32, e4582 (2023).

6. Greenfield, N.J. Using circular dichroism spectra to estimate protein secondary structure. Nat Protoc 1, 2876-90 (2006).

7. Wood, C.W. et al. BAlaS: fast, interactive and accessible computational alanine-scanning using BudeAlaScan. Bioinformatics 36, 2917-2919 (2020).

8. Suchyta, M., Miotto, B. & McGarry, T.J. An inactive geminin mutant that binds cdt1. Genes (Basel) 6, 252-66 (2015).
