# Supplementary material for: Geminin inhibits DNA replication licensing by sterically blocking CDT1-MCM2 interactions
Source: Nat Commun. 2025 Dec 9;16:11040. doi: 10.1038/s41467-025-67073-0 (PMC12695944; doi:10.1038/s41467-025-67073-0)
Supplement: Supplementary file 2 — Description of Additional Supplementary Files [file 41467_2025_67073_MOESM2_ESM.pdf]

Description of Additional Supplementary Files

File Name: Supplementary Data 1
Description: Additional information on oligonucleotides, recombinant DNA constructs and yeast strains used in this study.
