# Supplementary material for: Geminin inhibits DNA replication licensing by sterically blocking CDT1-MCM2 interactions
Source: Nat Commun. 2025 Dec 9;16:11040. doi: 10.1038/s41467-025-67073-0 (PMC12695944; doi:10.1038/s41467-025-67073-0)
Supplement: Supplementary file 4 — Reporting Summary [file 41467_2025_67073_MOESM4_ESM.pdf]

Corresponding author(s): Alexis Barr, Anna Barnard and Christian Speck

Last updated by author(s): 15th October 2025

Reporting Summary

Nature Portfolio wishes to improve the reproducibility of the work that we publish. This form provides structure for consistency and transparency in reporting. For further information on Nature Portfolio policies, see our Editorial Policies and the Editorial Policy Checklist.

Statistics

For all statistical analyses, confirm that the following items are present in the figure legend, table legend, main text, or Methods section.

n/a	Confirmed	
☐	☒	The exact sample size (*n*) for each experimental group/condition, given as a discrete number and unit of measurement
☐	☒	A statement on whether measurements were taken from distinct samples or whether the same sample was measured repeatedly
☐	☒	The statistical test(s) used AND whether they are one- or two-sided<br>*Only common tests should be described solely by name; describe more complex techniques in the Methods section.*
☐	☒	A description of all covariates tested
☐	☒	A description of any assumptions or corrections, such as tests of normality and adjustment for multiple comparisons
☐	☒	A full description of the statistical parameters including central tendency (e.g. means) or other basic estimates (e.g. regression coefficient) AND variation (e.g. standard deviation) or associated estimates of uncertainty (e.g. confidence intervals)
☐	☒	For null hypothesis testing, the test statistic (e.g. *F*, *t*, *r*) with confidence intervals, effect sizes, degrees of freedom and *P* value noted<br>*Give P values as exact values whenever suitable.*
☒	☐	For Bayesian analysis, information on the choice of priors and Markov chain Monte Carlo settings
☒	☐	For hierarchical and complex designs, identification of the appropriate level for tests and full reporting of outcomes
☒	☐	Estimates of effect sizes (e.g. Cohen's *d*, Pearson's *r*), indicating how they were calculated

Our web collection on statistics for biologists contains articles on many of the points above.

Software and code

Policy information about availability of computer code

Data collection	FujiFilm FLA-5100 Fluorescent Image Analyser and Multi Gauge V2.3 (FujiFilm) software. Statistical analysis was performed in GraphPad Prism 10, Thermo Fisher Talos F200i TEM, equipped with a Falcon 3EC direct electron detector, EPU software. AcquireMP software (v. 2023R2, Refeyn Ltd)
Data analysis	Multi Gauge V2.3 (FujiFilm), CryoSPARC (v4.6.2), DiscoverMP software (v2023R2, Refeyn Ltd), ASTRA 6 software (Wyatt Technology), LabSolutions software (Shimadzu), MassHunter Qualitative Analysis 10.0 software (Agilent), UCSF ChimeraX-1.9, Biacore Evaluation software (Cytiva), UCSF Chimera (1.17.3)

For manuscripts utilizing custom algorithms or software that are central to the research but not yet described in published literature, software must be made available to editors and reviewers. We strongly encourage code deposition in a community repository (e.g. GitHub). See the Nature Portfolio guidelines for submitting code & software for further information.

Data

Policy information about availability of data

All manuscripts must include a data availability statement. This statement should provide the following information, where applicable:

- Accession codes, unique identifiers, or web links for publicly available datasets
- A description of any restrictions on data availability
- For clinical datasets or third party data, please ensure that the statement adheres to our policy

Data that support the study are available from the corresponding authors upon reasonable request.

A source data file is available with the published article.

Research involving human participants, their data, or biological material

Policy information about studies with human participants or human data. See also policy information about sex, gender (identity/presentation), and sexual orientation and race, ethnicity and racism.

Reporting on sex and gender	N/A
Reporting on race, ethnicity, or other socially relevant groupings	N/A
Population characteristics	N/A
Recruitment	N/A
Ethics oversight	N/A

Note that full information on the approval of the study protocol must also be provided in the manuscript.

Field-specific reporting

Please select the one below that is the best fit for your research. If you are not sure, read the appropriate sections before making your selection.

[x] Life sciences [ ] Behavioural & social sciences [ ] Ecological, evolutionary & environmental sciences

For a reference copy of the document with all sections, see nature.com/documents/nr-reporting-summary-flat.pdf

Life sciences study design

All studies must disclose on these points even when the disclosure is negative.

Sample size	No statistical methods were used to predetermine sample size.
Data exclusions	Data was only excluded if there was a clear outlier within repeats of identical conditions.
Replication	Biochemical experiments were repeated at least two or three times as biological replicates.
Randomization	Experiments were not randomized due to the nature of the data not involving patients or animals. The experiments were not grouped and needed to be preformed in a particular order.
Blinding	Blinding is not applicable to this research, which does not entail randomization and does not involve patients or animals. The experiments required essential visual inspection of the data quality during data screening and data collection and so no blinding could be performed.

Reporting for specific materials, systems and methods

We require information from authors about some types of materials, experimental systems and methods used in many studies. Here, indicate whether each material, system or method listed is relevant to your study. If you are not sure if a list item applies to your research, read the appropriate section before selecting a response.

Materials & experimental systems

n/a	Involved in the study	
[ ]	[x]	Antibodies
[ ]	[x]	Eukaryotic cell lines
[x]	[ ]	Palaeontology and archaeology
[ ]	[x]	Animals and other organisms
[x]	[ ]	Clinical data
[x]	[ ]	Dual use research of concern
[x]	[ ]	Plants

Methods

n/a	Involved in the study	
[x]	[ ]	ChIP-seq
[x]	[ ]	Flow cytometry
[x]	[ ]	MRI-based neuroimaging

Antibodies

Antibodies used	Anti-CDT1 monoclonal mouse antibody (Santa Cruz #sc-365305, lot 2821,1:1000), Phospho-CDK Substrate Motif [(K/H)pSP] primary antibody (Cell Signaling Technology, #9477 lot 2, 1:1000), HRP-conjugated anti-mouse secondary antibody (Sigma A4416, 1:10000), anti-rabbit IgG HRP-linked secondary antibody (Cell Signaling Technology, #7074, 1:10000).

Validation	The antibodies are commercially available (product numbers included in methods), have been described in the literature and referenced in the manuscript.

Eukaryotic cell lines

Policy information about cell lines and Sex and Gender in Research

Cell line source(s)	HEK293 cells were used for overexpression of the hMCM2-7 complex, as reported previously in Rzechorzek, et.a., (2020), and were provided by Oxford expression technologies LTD.
Authentication	Cell line was not authenticated - used for the expression of purified protein (MCM2-7)
Mycoplasma contamination	Cell line was negative for mycoplasma.
Commonly misidentified lines (See ICLAC register)	*Name any commonly misidentified cell lines used in the study and provide a rationale for their use.*

Animals and other research organisms

Policy information about studies involving animals; ARRIVE guidelines recommended for reporting animal research, and Sex and Gender in Research

Laboratory animals	Xenopus laevis females for the collection of eggs
Wild animals	N/A
Reporting on sex	N/A
Field-collected samples	N/A
Ethics oversight	Work was performed under Home Office Project Licence PP5679614

Note that full information on the approval of the study protocol must also be provided in the manuscript.

Plants

Seed stocks	N/A
Novel plant genotypes	N/A
Authentication	N/A
